# Presence of 19 Mycotoxins in Human Plasma in a Region of Northern Spain

**DOI:** 10.3390/toxins12120750

**Published:** 2020-11-27

**Authors:** Beatriz Arce-López, Elena Lizarraga, Ángel Irigoyen, Elena González-Peñas

**Affiliations:** Pharmaceutical Technology and Chemistry Department, Research Group MITOX, School of Pharmacy and Nutrition, Universidad de Navarra, 31008 Pamplona, Spain; barce@alumni.unav.es (B.A.-L.); elizarraga@unav.es (E.L.); angelirigoyen@unav.es (Á.I.)

**Keywords:** human plasma, human biomonitoring, mycotoxins, ochratoxin A, ochratoxin B, sterigmatocistyn

## Abstract

This study was conducted to investigate human exposure to 19 compounds (mycotoxins and their metabolites) in plasma samples from healthy adults (n = 438, aged 19–68 years) from Navarra, a region of northern Spain. Samples were analyzed by LC-MS/MS, before and after enzymatic hydrolysis for the detection of possible glucuronides and/or sulfates (Phase II metabolites). The most prevalent mycotoxin was ochratoxin A (OTA), with an incidence of 97.3%. Positive samples were in the concentration range of 0.4 ng/mL to 45.7 ng/mL. After enzymatic treatment, OTA levels increased in a percentage of individuals, which may indicate the presence of OTA-conjugates. Regarding ochratoxin B, it has also been detected (10% of the samples), and its presence may be related to human metabolism of OTA. Sterigmatocystin was detected with a high incidence (85.8%), but only after enzymatic hydrolysis, supporting glucuronidation as a pathway of its metabolism in humans. None of the other studied mycotoxins (aflatoxins B1, B2, G1, G2 and M1; T-2 and HT-2 toxins; deoxynivalenol, deepoxy-deoxynivalenol, 3-acetyldeoxynivalenol, 15-acetyldeoxynivalenol; zearalenone; nivalenol; fusarenon-X; neosolaniol; and diacetoxyscirpenol) were detected in any of the samples, neither before nor after enzymatic treatment. To the best of our knowledge, this is the first report carried out in Spain to determine the exposure of the population to mycotoxins and some of their metabolites using plasma, and the obtained results justify the need for human biomonitoring and metabolism studies on mycotoxins.

## 1. Introduction

Under mycotoxin designation, a group of very heterogeneous compounds with different chemical structures, biochemical and physico-chemical characteristics, and also different toxicological properties is included [1]. These toxins are secondary metabolites produced by phytopathogenic fungi as *Aspergillus*, *Penicillium* and *Fusarium* [2]. In general, one fungal species can produce different mycotoxins, and also, some mycotoxins can be produced by more than one fungal species.

In addition to the free or unmodified forms of these toxins, other related compounds can be present in the human diet. For instance, their complexes with matrix compounds as proteins or polysaccharides (matrix-associated forms) and also toxins that have undergone a chemical or biological modification of their structure, e.g., formation of sulfates, glucosides or glucuronides by fungi, plants or animals as a result of metabolic processes (modified mycotoxins) [3,4].

Mycotoxins reach the human population through different exposure routes [5], being the most important one the ingestion through contaminated food [6]. It should be taken into account that most mycotoxins remain stable during food processing and even in human gastric juices with a low pH [7].

Mycotoxins present a severe hazard for human health as they can produce acute and chronic diseases. The main chronic toxicities caused by mycotoxins are neurotoxicity, nephrotoxicity, hepatotoxicity, genotoxicity, carcinogenicity, endocrine and reproductive disorders, immunotoxicity and oestrogenicity [5]. Other toxic effects have also been observed. These include mutagenicity, teratogenicity, protein inhibition, RNA and DNA synthesis, apoptosis and cellular necrosis, hematotoxicity, myelotoxicity, gastrointestinal problems, immune diseases and hyperestrogenic or feminizing syndromes [8].

The final effect of mycotoxins on human health is determined by several factors such as the type of toxin, its metabolism, pharmacokinetics and accumulation, time of exposure and excretion rates [9]. Moreover, the age, gender, immune system and health status of the exposed individual and the exposure conditions must be taken into consideration [10]. Besides, and due to the fact that different fungal species may appear simultaneously in the same food product in a varied human diet [11], co-exposure to several mycotoxins is a very plausible scenario and this could lead to additive, synergic or antagonistic toxic effects, although this last aspect is not well known [12]. Furthermore, exposure to modified mycotoxins poses an additional risk to human health because they may be as toxic or even more toxic than the parent compounds [13]. In addition, they may increase exposure to the parent compounds since they can be reconverted into free toxins during human metabolism [4].

The assessment of human exposure to mycotoxins can be carried out by analyzing the presence of the toxins directly in food matrices (external exposure), as well as indirectly through the analysis of adequate biomarkers (parent substances or their metabolites) in biological fluids or tissues (internal exposure) [14].

The measurement of external exposure has been extensively employed in the evaluation and assessment of the risks that mycotoxins pose to human health; although this approach faces a number of weaknesses. For instance, food preparation or individual health conditions could modify the bioavailability of toxins from the diet and consequently the exposure of the human body. Furthermore, the distribution of mycotoxins in food matrices is not homogeneous and accurate food consumption data are difficult to obtain [15].

In order to evaluate a real estimation of human exposure, internal analysis of mycotoxins in biological matrices through human biomonitoring (HBM) has been promoted as an indispensable complement to direct mycotoxin determination in food [14,16]. The World Health Organization defines HBM as “the method for assessing human exposure to chemicals or their effects by measuring these chemicals, their metabolites or reaction products in human specimens” [17]. HBM is a non-food dependent measure and provides a more accurate indicator of mycotoxin exposure, not only from oral source, but also from dermal and inhalation routes [18].

It is of the utmost importance to select the appropriate biomarkers for developing HBM of mycotoxins [19]. The measurement of the free forms or parent compounds is commonly used [20] but, in this case, no mycotoxins linked to matrix substances or biologically or chemically modified are detected [21]. Therefore, to avoid under- or overestimation exposure, it is advisable that most forms of mycotoxins (e.g., free forms, protein or DNA adducts or Phase I and Phase II metabolites, etc.) being examined [22]. 

In general, blood and urine are the most widely used matrices in human biomonitoring [1,20,23]. Each matrix has its own advantages and drawbacks, and the results obtained from the analysis of mycotoxins in both are complementary and useful in order to know the bioavailability, metabolism, toxicokinetics and toxicological characteristics of mycotoxins.

Their determination in plasma is a very useful tool. For some mycotoxins, levels found in plasma were higher than those in urine matrix and, therefore, their analyses in plasma require less sensitive methods. This is the case for ochratoxin A (OTA) [24] or citrinin [25]. In addition, the presence of some mycotoxins in plasma, e.g., OTA, represents long-term exposure [26].

HBM based on one-to-one evaluation of possible mycotoxins or metabolites using different methodologies is very costly and time-consuming. To optimize resources, the use of adequately validated methods for the multi-detection of mycotoxins in human fluids is needed [1,10,27]. For this reason, and for the purpose of carrying out appropriate HBM projects, our research group validated in 2020 a methodology able to determine 19 mycotoxins, free toxins and some metabolites, in human plasma samples. These compounds, classified in two groups for analytical purposes (see Section 5), are: group I: aflatoxins B1 (AFB1), B2 (AFB2), G1 (AFG1), G2 (AFG2) and M1 (AFM1); sterigmatocystin (STER); OTA and ochratoxin B (OTB); T-2 and HT-2 toxins; zearalenone (ZEA) and deepoxy-deoxynivalenol (DOM-1); and group II: deoxynivalenol (DON), deepoxy-deoxynivalenol (DOM-1), 3-acetyldeoxynivalenol (3-ADON), 15-acetyldeoxynivalenol (15-ADON), zearalenone (ZEA), nivalenol (NIV); fusarenon-X (FUS-X); neosolaniol (NEO) and diacetoxyscirpenol (DAS) [28].

In Navarra (a region of northern Spain), the presence of some mycotoxins has been investigated in previous studies in different food matrices, such as cereal products [29], wine [30], apple juice [31] and milk [32]. However, and to the best of our knowledge, no HBM data are available on the presence of multiple mycotoxins in plasma. Only one study was carried out in 1998 analyzing the presence of ochratoxin A in plasma samples from healthy volunteers [33].

In this paper, we present the results obtained regarding the exposure to 19 compounds (mycotoxins and some of their metabolites) in healthy individuals from Navarra based on their analysis in plasma samples. Plasma samples have been analyzed before and after the treatment with a mixture of β-glucuronidase and arylsulfatase in order to study, in an indirect way, the presence or not of glucuronide or sulfate metabolites of the studied mycotoxins. 

## 2. Results

### 2.1. Control of the Analytical Sequences

Plasma samples were analyzed in groups or sequences in which matrix-matched calibrators were included. In this way, the levels of each mycotoxin in each sample were calculated using the calibration curve obtained from calibrators in the same sequence. Each one of the calibration curves employed in mycotoxin quantification fulfilled the criteria defined during the validation of the method: a minimum of six points, a determination coefficient > 0.99, and back-calculated mycotoxin concentration for each one of the calibration samples not differing by more than 15% from the nominal value (20% for limit of quantification (LOQ)). An example of the obtained calibration curves for each mycotoxin is presented in Table S1. In addition, for each one of the mycotoxin peaks detected, the following criteria have been accomplished: both, qualification (q) and quantification (Q) transitions were present, and with a q/Q ratio (in percentage) similar to the mean value obtained for this mycotoxin in calibrators (Table 1). In each one of the individual samples, the obtained relative error (RE) between q/Q ratio values in calibrators and in samples was less than 20%.

Additionally, retention times in each one of the individual samples and the mean value obtained in calibrators did not differ by more than 2.5% for OTA before and 1.4% after enzymatic treatment. In the case of OTB, 1.8% and 0.4%, respectively, and 0.5% for STER (Table 2).

### 2.2. Re-Validation of the Methodology after Enzymatic Treatment

Recovery values obtained for all the mycotoxins after enzymatic treatment were from 73.7% for NIV to 90.1% for HT-2 (RDS ≤ 15% for all of them). The recovery level obtained for each mycotoxin was within the range calculated during the method validation using plasma samples without enzymatic treatment [28]. Therefore, the enzymatic treatment did not influence this parameter. In the case of matrix effect (ME), values ranged between 71.2% (DOM-1) to 105.8% (T-2) (RSD ≤ 15% for all of them). In this parameter only DOM-1 and AFG2 obtained a slightly lower ME value than that obtained using plasma samples before enzymatic treatment. Limits of detection (LODs) and LOQs maintained the same values as those obtained without enzyme treatment. Details regarding data obtained during method re-validation are provided in the Supplementary Data (Tables S2 and S3).

### 2.3. Plasma Samples

**Figure 1:**
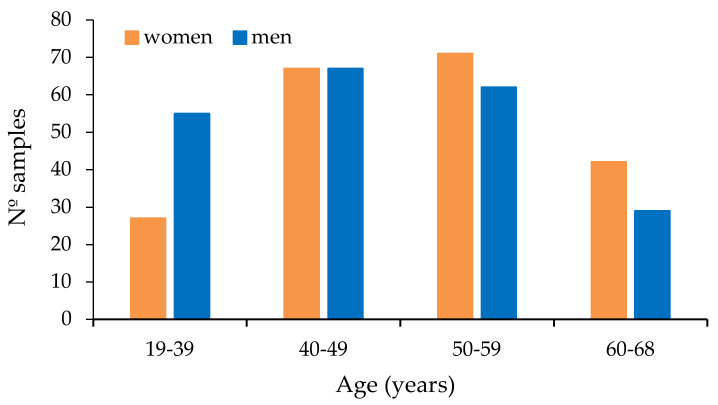
Distribution of donors according to age.

Four hundred and thirty-eight samples were collected from 438 different and healthy donors. Out of 18 donors, no age or gender data was recorded. The other 420 participants were between 19 and 68 years old. Women had a mean age of 50.6 ± 9.4 years and men of 46.9 ± 11.2 years. The global mean was 48.7 ± 10.5 years. In Figure 1, the distribution of donors according to their age is shown. There are significant differences between both genders (*p* = 0.0012, 95% confidence interval (CI)), with men donors being slightly younger than women. Nearly fifty percent of the samples (207) were from women and 213 from men.

### 2.4. Chromatographic Results

**Figure 2:**
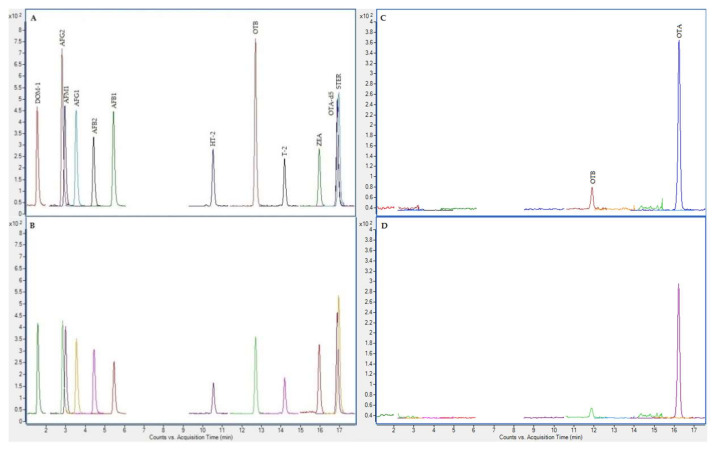
Superposed extracted chromatograms obtained for mycotoxins group I from a calibrator at 10 × LOQ level (**A**,**B**) and a plasma sample (number 47) (**C**,**D**) before enzymatic treatment. (**A**,**C**) display the quantification transition, and (**B**,**D**) the qualification transition, respectively.

**Figure 3:**
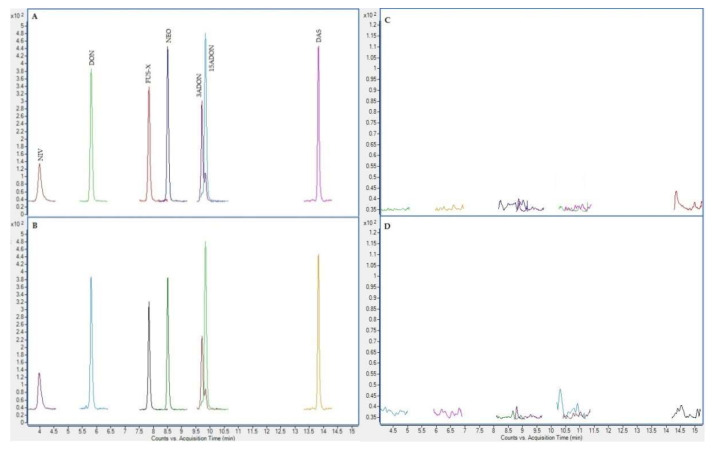
Superposed extracted chromatograms obtained for mycotoxins group II from a calibrator at 10 × LOQ level (**A**,**B**) and a plasma sample (number 47) (**C**,**D**) before enzymatic treatment. (**A**,**C**) display the quantification transition, and (**B**,**D**) the qualification transition, respectively.

**Figure 4:**
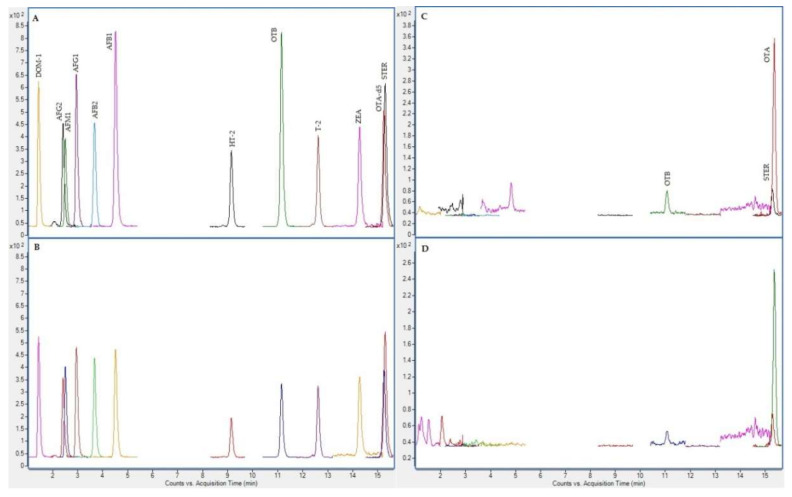
Superposed extracted chromatograms obtained for mycotoxins group I from a calibrator at 10 × LOQ level (**A**,**B**) and a plasma sample (number 213) (**C**,**D**) after enzymatic treatment. (**A**,**C**) display the quantification transition, and (**B**,**D**) the qualification transition, respectively.

**Figure 5:**
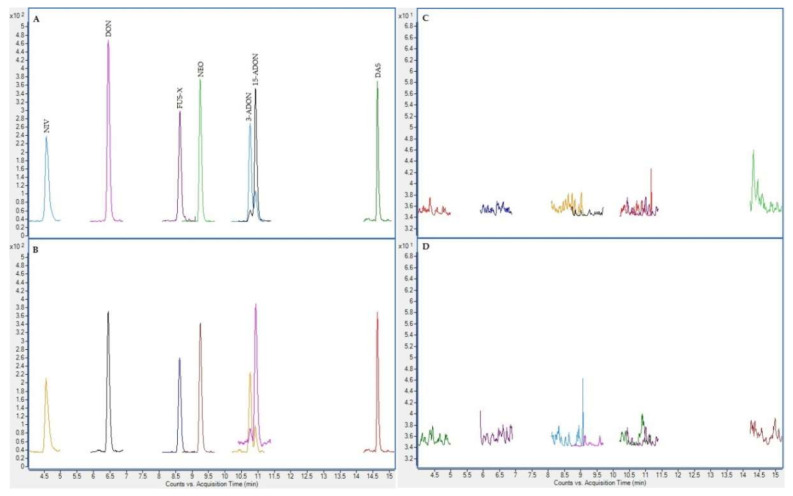
Superposed extracted chromatograms obtained for mycotoxins group II from a calibrator at 10 × LOQ level (**A**,**B**) and a plasma sample (number 213) (**C**,**D**) after enzymatic treatment. (**A**) and display the quantification transition, and (**B**,**D**) the qualification transition, respectively.

In the next figures (Figure 2, Figure 3, Figure 4 and Figure 5), superposed extracted chromatograms obtained from calibrators and samples, before and after enzymatic treatment, are shown.

### 2.5. Mycotoxins in Samples

In the following Table 3, Table 4 and Table 5, the results obtained for positive samples before and after enzymatic treatment are shown (values < LOD are not indicated). In Table 3, results obtained on those samples without data available on gender and age are presented. In Table 4 and Table 5 results in samples of women and men, respectively, are included. The age of the donors is also indicated in both tables.

#### 2.5.1. Reanalysis

The 70% of the reanalyzed samples fulfilled the FDA guideline criteria (RE no more 20%) for OTA levels (Table S4), a higher percentage than that fixed (67%) [34]. Therefore, the analysis was accepted. After enzymatic treatment, no reanalysis was possible because there was not a sufficient plasma volume.

#### 2.5.2. Results before Enzymatic treatment

The results obtained before enzymatic treatment are summed-up in Table 6 and Table 7 and they can be described as follows. Among the 19 mycotoxins under study, 426 plasma samples were found to contain OTA at detectable levels (> 0.4 ng/mL). Forty-four plasma samples contained OTB at levels above its LOD (0.4 ng/mL). DOM-1, AFG2, AFM1, AFG1, AFB2, AFB1, ZEA, STER, T2, HT-2, DON, FUS-X, NEO, 3-ADON, 15-ADON or DAS were not detected in any of the analyzed samples at the LODs achieved with the method.

From these data, it is deduced that the most prevalent mycotoxin in human plasma samples in Navarra was OTA, which presented values higher than its LOD (0.4 ng/mL) in the range from 0.4 ng/mL to 45.7 ng/mL. Besides, OTB was also detected in the range from LOD (0.4 ng/mL) to 1.7 ng/mL. Three hundred and eighty-two samples contained only OTA, and 44 samples contained OTB. Interestingly, all the samples in which OTB was detected also showed detectable levels of OTA.

Regarding gender distribution, in women, OTA levels > LOD were found in 197 plasma samples and between 0.4 and 15.6 ng/mL, although one sample (sample 47) reached 45.7 ng/mL. This last value was the maximum level encountered in all the analysed plasma samples and came from a 66-year-old donor. In this woman, the highest level of OTB was also encountered (1.7 ng/mL). This plasma sample was reanalyzed obtaining the following values: 42.3 and 1.4 ng/mL for OTA and OTB, respectively. In both cases, a RE (%) below 20% was obtained, as stated by the FDA in regard to the incurred sample reanalysis [34].

For men, the range was from < LOD to 19.9 ng/mL and OTA presented > LOD levels in 211 samples. The highest level of OTA came from a 56-year-old donor (sample 512, 19.9 ng/mL), and in this sample, OTB was also present at 0.9 ng/mL.

**Figure 6:**
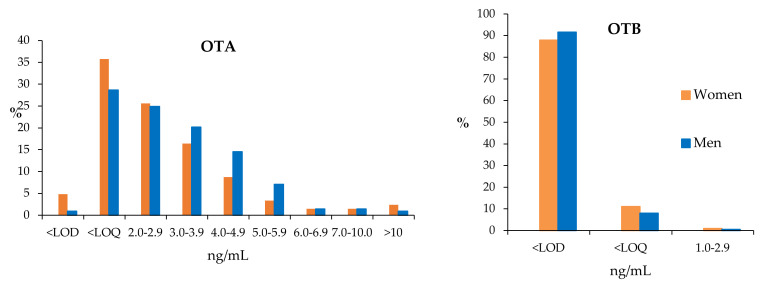
Percentage of positive samples for OTA and OTB, before enzyme treatment, at different range levels (ng/mL).

In order to clarify data distribution, the interval of the total OTA and OTB levels has been divided into different ranges of plasma concentrations (ng/mL), and in each one of them, the percentage of positive samples regarding OTA and OTB has been calculated. The results are shown in Figure 6.

Figure 6 shows a tendency for women to have a higher incidence than men in OTA values < 3 ng/mL and in samples with high levels of contamination (> 10 ng/mL).

Following a Wilcoxon test, significant differences were found between men and women in OTA levels (*p* < 0.05, 95% CI), even without taking into account the higher OTA level in one-woman sample. Men have higher % positive values and mean and median values.

For OTB, no significant differences were found between men and women (*p* = 0.63, 95% CI). Of women samples, 99.0% have OTB values below their LOQ, whereas in men the percentage is 99.5%.

**Figure 7:**
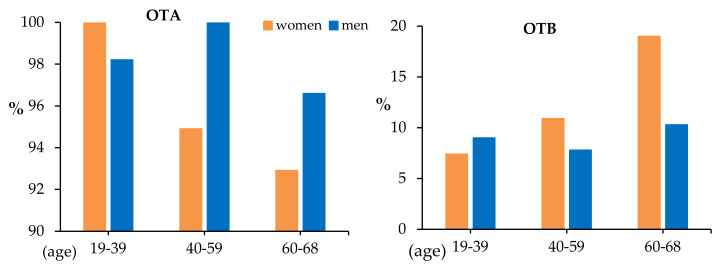
Incidence of OTA and OTB in each one of the age intervals (years).

With regard to age groups, Figure 7 shows the incidence of OTA and OTB according to the age of donors (years).

As can be seen in Table 6 and Table 7, no statistical differences in OTA or OTB levels between age groups have been found. However, OTA incidence tends to diminish with increasing age in women, whereas in men, the incidence is more stable in the three age intervals (Figure 7). For OTB, incidence increased as the age of women increased (in contrast with the trend in OTA incidence), while for men, the incidence tends to be stable in the three age intervals (similar, but complementary, to the trend in OTA incidence) (Figure 7).

#### 2.5.3. Results after Enzymatic Treatment

After the enzymatic treatment, 346 plasma samples were analyzed, 180 from men and 166 from women. The results are shown in the following tables (Table 8, Table 9 and Table 10), and they can be summed-up as follows. Among the 19 mycotoxins under study, 323 plasma samples were found to contain OTA at detectable levels (>0.4 ng/mL). Forty-seven plasma samples contained OTB at levels above its LOD (0.4 ng/mL). In contrast to the results obtained before enzymatic treatment, STER was also detected in 297 out of 346 plasma samples at levels above its LOD (0.2 ng/mL). No other mycotoxins were found in samples at the LODs achieved by the present method.

The most prevalent mycotoxin was once again OTA, which presented values above its LOD (0.4 ng/mL) in the range from 0.4 ng/mL to 23.3 ng/mL (Table 8). Besides, OTB was also detected in the range from < LOD (0.4 ng/mL) to 1.3 ng/mL (Table 9). STER appeared in the range from 0.2 to 1.4 ng/mL in 144 women and 153 men plasma samples (Table 10).

**Figure 8:**
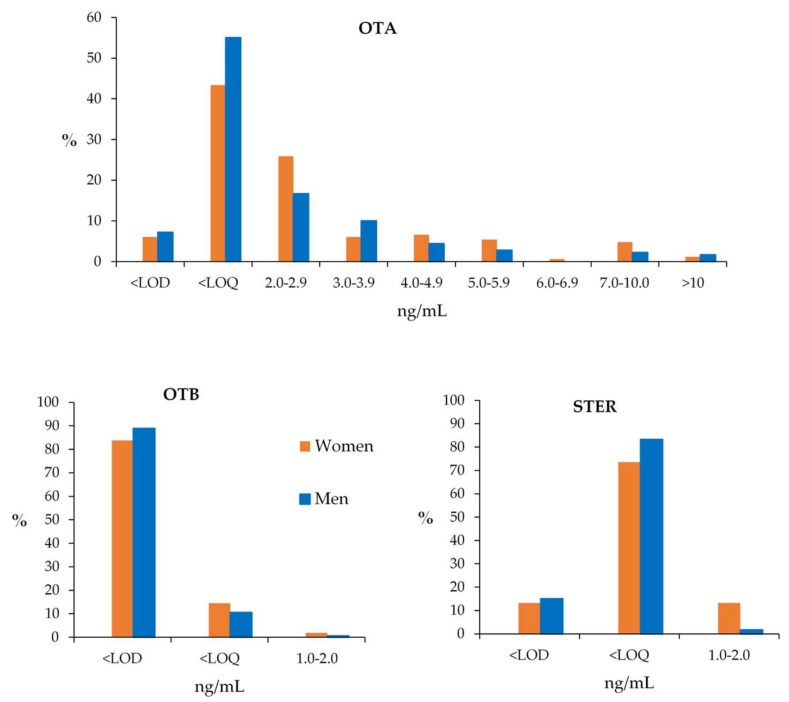
Incidence of OTA, OTB and STER after enzyme treatment at different range levels (ng/mL).

The incidence at each of the mycotoxin range levels (ng/mL) regarding OTA, OTB and STER in the 346 samples is shown in Figure 8.

**Figure 9:**
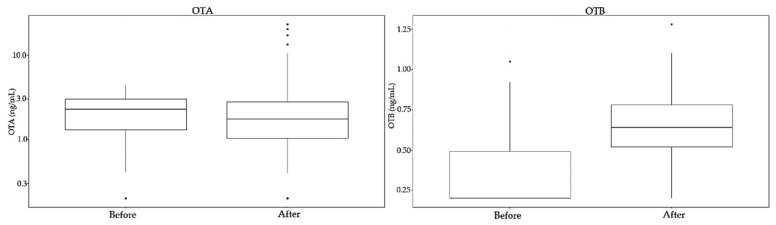
Comparison between total OTA (left) and OTB (right) levels before and after enzymatic treatment.

**Figure 10:**
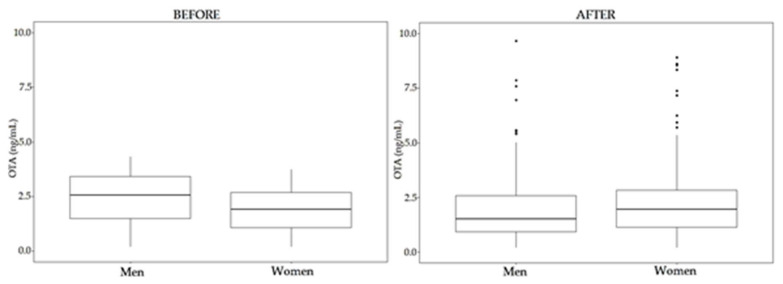
Comparison between OTA levels before (left) and after (right) enzymatic treatment, according to gender.

Regarding OTA, once again significant differences have been observed between women and men samples after enzymatic treatment (*p* < 0.05, 95% CI). Moreover, statistical differences have been observed between the levels in the samples before and after enzymatic treatment (*p* < 0.05, 95% CI) (Figure 9). These differences are due to the group of men samples (*p* < 0.05 between samples before and after enzymatic treatment); while no significant differences have been observed between OTA levels from women samples (*p* = 0.11, 95 % CI) (Figure 10). Considering individual samples, 58.4% of women samples and 29.4% of men samples show an increase in OTA concentration after enzymatic treatment.

For OTB, significant differences have been seen in the levels of this mycotoxin before and after enzymatic treatment (*p* < 0.05, 95% CI) (Figure 9), also due to men samples (*p* < 0.05, 95% CI). In the case of women samples, enzymatic treatment did not give to significant differences (*p* = 0.15, 95% CI).

Regarding age groups, there are not significant differences between them, neither in the case of OTA nor OTB (see Table 8 and Table 9).

**Figure 11:**
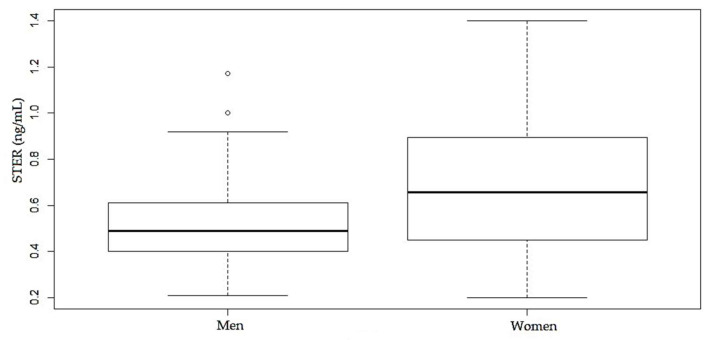
Comparison between STER levels after enzymatic treatment, according to gender.

In the case of STER, significant differences have been observed between the levels of women and men samples (*p* < 0.05, 95% CI) (Figure 11). Women have higher values of % positives and mean and median values. Regarding age groups, the incidence of the STER (%) is stable with the age of donors, both in women and men groups, and there are not significant differences between them (*p* < 0.05, 95% CI).

Overall, in twenty-nine samples (8.4%), the 3 mycotoxins co-occurred, and all samples in which OTB was detected also showed detectable levels of OTA.

## 3. Discussion

HBM is an interesting and challenging approach applied in the study of human exposure to mycotoxins. This approach has advantages, as indicated in the introduction section of this paper. However, HBM of mycotoxins faces some challenges such as the description of good biomarkers and the relationships between their levels in human fluids or tissues and human risk, the necessity to increase the general knowledge about the metabolism and toxicokinetics of these compounds or to develop adequate and validated analytical methodologies, among others. Nevertheless, HBM is also a great opportunity for improving risk assessment because it is recognized as an important tool to estimate the real human exposure to toxicants [22].

A number of studies have been carried out on the HBM of mycotoxins in human blood, serum or plasma, either through single-biomarker studies (analysis of one mycotoxin or some related mycotoxins) or multi-biomarker studies (analysis of multiple mycotoxins in a single run) [3,10,14,35,36,37]. This last approach is the preferable one, with the further aim of saving money and time.

In a recent review published by our research group [1], the state-of-the-art on HBM of mycotoxins in plasma, serum and blood samples was summarized. In that study, it was concluded that AFB1 (as AFB1—lysine adducts) and OTA were the most widely examined and detected human biomarkers for mycotoxins in recent years. Aflatoxins (AFB1, AFB2, AFG1, AFG2 and AFM1), citrinin, STER and ZEA were also analyzed, but to a much lesser extent. Other mycotoxins such as T-2 and HT-2 were not studied. Phase II metabolites, such as sulfates, glucosides or glucuronides derivatives were not detected in plasma samples, although the applied analytical methodologies included their detection [38,39].

In the present work, the occurrence of a total of 19 compounds, including mycotoxins and some of their metabolites, has been studied for the first time in 438 human plasma samples from Navarra, Spain. Among the 19 analytes investigated, mycotoxins of major risk for human health, such as OTA, or aflatoxins have been studied. In addition, some related compounds such as OTB or DON metabolites, and mycotoxins rarely studied in human plasma, such as trichothecenes, T-2, HT-2 or STER, have been included. Finally, the presence of Phase II metabolites (glucuronides and sulfates) has been also examined. To the best of our knowledge, no HBM data are available on the presence of multiple mycotoxins in plasma in Navarra nor in Spain.

The analysis of the plasma samples detected mainly OTA, which appeared in 97.3% of the samples in a range from <LOD to 19.9 ng/mL, although one woman sample reached a very high level of 45.7 ng/mL. In a study carried out in 1998 by our research group and after analyzing the presence of ochratoxin A in plasma samples from 75 healthy volunteers, OTA was detected in 53.3% of the samples in a range between <LOD (0.52 ng/mL) and 4 ng/mL, with a mean value of 0.71 ng/mL [33]. These results, with values much lower than those obtained in the present study, are indicative of an increase of human exposure to ochratoxin A in this region.

In some Spanish regions, different studies have been developed on the presence of OTA in human plasma, serum and blood samples, as can be seen in Table 11. All of them were carried out before 2011, and in almost all of the cases, the percentage of positive samples was high and similar to that found in the present study, although lower mean and maximum levels were reported.

As regards OTA HBM studies carried-out worldwide, Soto et al. (2016) [45] and Fromme et al. (2016) [46], published reviews covering until 2015. The incidence of OTA in plasma samples was from 35%–100% (in Spain close to 100%) [45]. The maximum level found was 74.8 ng/mL in Argentina [45].

OTA HBM studies carried-out in plasma samples from healthy volunteers during the last five years are shown in Table 12. As it can be observed, the incidence is high and similar to that obtained in the present study; nevertheless, lower OTA levels have been reported.

In Navarra, the presence of OTA in different food matrices (cereals and derived products, wine, beer and milk) has been studied. Levels of OTA have been found, but in most of the cases below the maximum legislated for the European Union in these matrices: 2 ng/mL for wine, 5 µg/kg for cereals, 3 µg/kg for processed cereals and 0.5 µg/kg for baby food cereals [60]. Only one sample of corn and two samples of baby food cereals exceeded their maximum limits. In one of these studies, OTB and ochratoxin C (OTC) have been also found in 100% and 70.6% of the wine samples analyzed (from 0.003 to 0.070 ng/mL and from <LOD to 0.014 ng/mL), respectively [30]. The results of these studies are summed up in Table 13.

A contradictory situation appears in this region, as can be deduced from the above data: low levels in food although higher levels in plasma than those reported in other regions or countries. Although OTA levels in plasma have been recognized as a good biomarker of dietary exposure to OTA [65], no correlation between OTA levels in food and in plasma has been observed for some authors [40]. In fact, many uncontrolled factors can affect the estimation of the real exposure using both, external and internal methodologies [66]. One factor that should be taken into account is that the analyzed matrices in Navarra were mainly those for which maximum levels have been legislated and, for this reason, also routinely controlled. Therefore, other OTA sources should be of importance in this region, for instance: chili pepper, preserved meat, cheese, grains and grain-based products, dried and fresh fruits, coffee, pork and spices, among others [48,65]. Furthermore, if only levels of OTA in a food product are analyzed, human exposure to this mycotoxin can be underestimated. For example, it is described that OTC is rapidly converted to OTA after oral administration in rats [65], and OTC has been found in wine [30]. Besides, it should be noted that OTA could also reach humans by inhalation and/or skin contact [5]. In addition, data on OTA metabolism and toxicokinetics should be increased in order to achieve a better estimation of the relationship between plasma and food levels for this mycotoxin.

The association between OTA levels in plasma and estimated daily intake (EDI) (*k*_0_, ng OTA/kg bw/day) has been described in the literature using the Klaassen equation (Equation (1)).

*k*_0_ (ng/kg.bw/day) = (*Cl*_renal_ × *C*p)/A (1)

In which, *Cp* refers to the OTA plasma level (ng/mL); A refers to OTA bioavailability (believed to be 0.5); body weight (bw) (assumed to be 70 kg); and *Cl*_renal_ is the daily renal clearance (mL/kg bw/day). For this last parameter, values of 0.048 mL/min or 0.1099 mL/min [47] have been proposed.

In case of choosing *Cl_r_*_enal_ = 0.1099 mL/min, the Klaassen equation can be expressed as Equation (2):

*EDI* = 4.52 × *C*p (2)

For OTA, a Tolerable Weekly Intake (TWI) value of 100 ng/kg.bw per week [67] or 120 ng/kg.bw per week [68] has been established. These values correspond to 14 or 17 ng/kg.bw per day, respectively, and the corresponding OTA levels in plasma, derived for Equation (2), are 3.1 and 3.8 ng/mL. From the data obtained in the present study, 33.6% of the individuals surpass these values (21.7% of women and 25.4% of men). Moreover, and with regard to OTA carcinogenicity, EFSA has established that the TWI of 120 ng/kg bw is no longer valid, and it is necessary to apply a margin of exposure approach for the characterization of the risk of OTA to human health [65]. For these reasons, human exposure to OTA in this region would be of concern.

Regarding gender, significant differences have been found among OTA levels in women and men, being higher in men samples. This result is in accordance with that found in different studies [45], although this aspect is not well known and there are contradictory results [48]. Fan et al. (2020) [48] found no significant differences between OTA levels in both genders; however, OTA levels were slightly higher in men. In the study of Warensjö et al. (2020), OTA serum concentration did not differ between both genders in Swedish adolescents [47], and Coronel et al. (2011) found no differences in plasma OTA levels between genders [40].

As for age-related OTA in plasma, overall and in different studies, OTA levels increased as age of donors increased [40]; however, there is no clear correlation between those variables [45]. Warensjö et al. (2020) found higher levels in OTA serum levels in the youngest Swedish adolescents [47]. In the present study, no differences in OTA levels have been found after dividing the samples into three age ranges (19–39, 40–59 and 60–68 years old); however, and in relation to incidence of OTA (% positives), a different situation has been observed in men and women. Whereas in women the incidence tends to decrease as the age of donors increases, in men this value remains similar in the three age groups (see Figure 7).

OTB, the dechlorinated form of OTA, has also been found in human plasma samples from Navarra. To the best of our knowledge, this is the first time that this mycotoxin has been detected in this matrix. This toxin, with lower toxicity than OTA [46], has not usually been included in HBM studies on mycotoxins. Nevertheless, both mycotoxins co-occurred in the plasma samples analyzed in this study.

On the one hand, the presence of OTB has been described in some food products such as wine [30]; and on the other, this mycotoxin has been also considered a metabolite of OTA “in vitro” and “in vivo” studies, but this last aspect has not been elucidated. This is due to the fact that the design of the studies that have been carried-out did not discriminate against OTB resulting from OTA metabolism or OTB that was present as a contaminant of the employed OTA [65]. However, “in vitro” studies on human liver microsomes have evidenced a high capability of dechlorination in humans [69].

In the present study, no correlation has been found between OTA-OTB levels in plasma (Spearman r = −0.13, *p* = 0.41 for total samples; r = −0.19 and *p* = 0.37 for women samples and r = −0.13 and *p* = 0.62 for men samples). However, it should be noted that the obtained Spearman correlation coefficients have negative values; OTB only appears in samples where OTA has been detected, and the incidence of OTB related to the age of donors increases with the age of women (contrary to the trend observed for OTA), whereas in the case of men it remains similar across age groups (similar and complementary to the trend observed for OTA). These observations may suggest that OTB appears in the plasma samples as an OTA metabolite. However, more studies on OTA human metabolism should be carried out in order to clarify this aspect.

DOM-1, AFG2, AFM1, AFG1, AFB2, AFB1, ZEA, T2, HT-2, NIV, DON, FUS-X, NEO, 3-ADON, 15-ADON and DAS levels were not detected, even after enzymatic treatment.

The non-detection of aflatoxins in samples is in accordance with HBM studies conducted in Europe, which indicated that aflatoxins are not the predominant mycotoxins to which the European population is exposed [18], or with Al-Jaal et al. (2020) [35] who did not identify aflatoxins in any plasma samples from Qatari donors. However, De Ruyck et al. (2020) in a European multi-center study found a prevalence of aflatoxins in 57% of the serum samples from 600 European individuals [66]. These authors studied aflatoxins (AFB1, B2, Q1, G1, G2 and M1) achieving LODs for these compounds in the range of 0.001–0.005 ng/mL, much lower than those obtained in the present study or that obtained by Al-Jaal et al. (2020) for these compounds (0.1 ng/mL) [35]. Furthermore, De Ruyck et al. (2020) also included the AFB1-lysine adduct in their determination. These considerations should be taken into account in order to explain the different results obtained in the detection of aflatoxins in human plasma/serum samples.

The non-detection of other mycotoxins or metabolites in plasma could be due to a low human exposure to them, but also, factors such as rapid metabolism and/or excretion through the urine, which is one of the main excretion route of toxicants [70], should be considered. For instance, Vidal et al. (2018) [71] found that the 74% of the administered dose of DON was recovered in human urine within 24 h. In addition, Fan et al. (2019) described higher levels in urine than in plasma for fumonisin B1 and zearalanone [38].

After enzymatic hydrolysis, OTA was, once again, the most prevalent mycotoxin, and its levels increased in some individuals after this treatment (58.4% of women and 29.4% of men samples). The effect of the enzymatic treatment is more relevant for women. In order to explain these results, interindividual and gender variations should be considered with respect to differences in enzyme activity of Phase II metabolism. However, due to the treatment, no significant differences have been found in the women group. On the contrary, significant differences have been observed for men, with lower mean and median values after hydrolysis.

Some authors, such as Fan et al. (2020), detected higher levels of OTA after treatment with β-glucuronidase and arylsulfatase in blood plasma samples in China. They concluded that this was due to the presence of OTA conjugates [48]. The results of the present study, with increased OTA levels in some individuals after hydrolysis (>50% in women), may also support the formation of OTA conjugates in the human metabolism of this mycotoxin.

In the literature, there are contradictory data regarding the formation or not of OTA-glucuronides and/or sulfates during the metabolism of OTA in humans. These OTA conjugates have been found in animal tissues and in human urine [70]; however, glucuronidated forms have not yet been identified [27]. EFSA considers that, although formation of conjugates with glucuronic acid and sulfate in OTA metabolism has been considered, the major metabolic route is its hydrolysis to OTα, followed by conjugation of this OTA derivative with glucuronic acid [65]. It is clear that more studies on OTA metabolism in humans need to be conducted.

OTB has also been found in plasma samples after enzymatic treatment, and no differences relating to gender or age of donors have been observed.

It is remarkable that STER only appeared in samples after being submitted to the enzymatic treatment with β-glucuronidase/arylsulfatase. This mycotoxin, a precursor in the biosynthesis of aflatoxins, has been classified as Group 2B (possible carcinogen for humans) by the IARC [72]. STER can be found in cheese, cereals, spices, beer, bread and even in housing or building materials [73]. Little is known about its metabolism, although glucuronidation is probably the main pathway, as demonstrated in animal urine [27]. In Qatar, STER has been detected in a lower percentage (10.9% of the plasma samples) than in the present study, perhaps because these authors did not use enzymatic treatment; although the concentration range found was quite similar, from 0.3 to 1.4 ng/mL [35]. Cao et al. (2018) detected STER in plasma samples after enzymatic treatment using β-glucuronidase [49]. The present study supports the formation of STER-glucuronides in human plasma samples. Moreover, STER appeared in a high percentage of samples and significant differences have been observed between the levels from women and men samples (*p* < 0.05, 95% CI), but not in relation to the age of the donors (*p* = 0.48, 95% CI).

## 4. Conclusions

This study reported data obtained after the analysis of 19 mycotoxin biomarkers in plasma samples from healthy human volunteers in a region of northern Spain (Navarra). For the first time, and within a human biomonitoring study, exposure to multiple mycotoxins and their metabolites of the population living in Navarra has been assessed. OTA is the prevalent mycotoxin that has been found, at levels that could indicate a human health concern. It is necessary to increase the knowledge of its toxicokinetics, the interindividual differences related to age and gender and their metabolism in humans to establish adequate relationships between plasma levels and risk to human health. Moreover, the quantification of OTA levels in less tested food matrices and also the study of additional sources of human exposure in this region should be carried-out in order to have greater control over them and to minimize the risk to humans. OTB appeared in human plasma samples and in all cases in relation to the presence of OTA, but the reason for this co-occurrence is not clear. OTB could be present in the human diet or be produced during OTA human metabolism or both scenarios. However, data from this study would indicate a relationship between the presence of OTB in plasma and OTA metabolism.

STER has also been detected in the analyzed plasma samples but only after enzymatic hydrolysis, enforcing the thesis that glucuronidation is one of the pathways of STER human metabolism. Due to the toxicity of this compound, further studies should be considered in order to identify the sources of human exposure and its possible metabolism pathway.

Other studied mycotoxins: DOM-1, AFG2, AFM1, AFG1, AFB2, AFB1, ZEA, T2, HT-2, NIV, DON, FUS-X, NEO, 3-ADON, 15-ADON and DAS have not been detected in any of the samples, neither before nor after enzymatic treatment.

HBM studies on mycotoxins should continue, along with the analysis of mycotoxins in food. Both are different, but complementary, approaches to obtain new data to better assess the hazard that mycotoxins pose to human health.

## 5. Materials and Methods

### 5.1. Subject Recruitment

Donors were healthy women and men, volunteers, who were habitual contributors to the Blood and Tissues Bank in Navarra, a region in northern Spain. From all of them, written informed consent was obtained for their participation, and the procedure was approved by Ethical Committee of the University of Navarra and the Blood and Tissue Bank of Navarra (015/2012) on 16 February 2012. Participants in this study gave only their gender and age as personal information. Blood samples (n = 438) were obtained during the year 2013.

### 5.2. Plasma Sample Collection

Five mL of blood was obtained from each participant. The sample was collected in 5 mL BD Vacutainer^®^ Plasma Tubes (Madrid, Spain) with EDTA as anticoagulant. Plasma was obtained after blood centrifugation at 12,000 × *g* for 10 min at 4 °C. The resulting plasma from each volunteer was separated into two tubes and frozen at −80 °C until analysis. Previous to the mycotoxin analysis, plasma was thawed and vortexed during a few seconds.

### 5.3. Sample Analysis

Analysis of mycotoxins in plasma samples before enzymatic treatment was carried out following the method reported by Arce-López et al. (2020) [28]. Briefly, chromatographic separation and detection were achieved in an LC system 1200 series coupled to a 6410 Triple Quadrupole (QqQ) in ESI (+) mode, both from Agilent Technologies (Waldbronn, Germany). An Ascentis Express C18, 2.7 μm particle size 150 × 2.1 mm column (Supelco Analytical, St. Louis, MO, USA) at 45 °C was used. Mobile phase was a mixture formed by solution A: 5 mM ammonium formate and 0.1% formic acid in water, and solution B: 5 mM ammonium formate and 0.1% formic acid in 95:5 methanol/water. Chromatographic separation was in gradient conditions. Flow rate was of 0.4 mL/min and volume of injection 20 μL. Data acquisition parameters are those described in Arce-López et al. (2020) [28]. Using this methodology, 19 mycotoxins and metabolites can be quantified. For analytical purposes, they were classified in two groups: group I included DOM-1, AFG2, AFM1, AFG1, AFB2, AFB1, OTB, ZEA, STER, OTA, T2, HT-2. Mycotoxins included in group II were NIV, DON, FUS-X, NEO, 3-ADON, 15-ADON and DAS. This classification was needed because each group was chromatographed using a different elution program in two different chromatographic analyses.

The method for plasma treatment before enzymatic treatment achieved the simultaneous extraction of all the studied mycotoxins and was also described in Arce-López et al. (2020) [28]. Concisely, it was as follows: 0.4 mL of human plasma were added to a Captiva EMR-lipid cartridge that contained 1.2 mL of acetonitrile acidified with formic acid at 1%. After 5 min, vacuum was applied, and the effluent was divided into two 0.4 mL portions. Each one of them was put in one tube and evaporated until dry (60 °C). In one of the tubes, 200 μL of 40% B-mobile phase were added to redissolution of mycotoxins group I. The second tube was reconstituted with 200 μL of 5% B-mobile phase and was employed for analyzing mycotoxins classified as group II.

This methodology was successfully validated following the FDA and EMEA guidelines for bioanalytical method validation. LOD values, calculated using a signal/noise ratio of 3 for the least sensitive transition, were 1.35 ng/mL for DOM-1, 0.35 ng/mL for AFG2, 0.18 ng/mL for AFM1, 0.07 ng/mL for AFG1 and AFG2, 0.04 ng/mL for AFB1, 2.70 ng/mL for HT-2, 0.40 ng/mL for OTA and OTB, 0.20 ng/mL for T-2 and STER, 1.80 ng/mL for ZEA, 9.10 ng/mL for NIV, 1.94 ng/mL for DON, 1.95 ng/mL for FUS-X, 0.18 ng/mL for NEO, 0.70 ng/mL for 3-ADON, 1.20 ng/mL for 15-ADON and 0.15 ng/mL for DAS. Recovery, obtained in intermediate precision conditions, was in the range of 68.8% for STER to 97.6% for DAS (RDS ≤ 15% for all the mycotoxins). Matrix effects were also evaluated and were not significant for most of the mycotoxins with RDS values ≤ 15% for all of them.

To account for the possible presence of Phase II metabolites (glucuronide and sulfate conjugates), a set of plasma samples (n = 346) was enzymatically treated with β-glucuronidase/arylsulfatase (from Helix Pomatia, Sigma Aldrich, Mannheim, Germany) prior to further sample clean-up. The treatment was as follows: 50 µL of β-glucuronidase/arylsulfatase enzyme (250 U/mL, 0.2 U/mL in PBS) were added to 400 µL of plasma. After agitation, samples were incubated in water bath overnight (37 °C). Then, enzymatically treated plasma samples were processed as described above.

The chromatographic analysis of the enzymatically treated samples was made following the procedure described above. However, a re-validation of the methodology was carried out in order to check the influence of the enzymatic treatment in the obtained quantification results. The procedure and criteria employed for re-validation were those described in Arce-López et al. (2020) [28]. For this purpose, fortified plasma samples that were enzymatically treated were used. Precision and accuracy were evaluated at three different levels (LOQ, 6 × LOQ and 30 × LOQ) in triplicate and in between-day (3 days) conditions. Recovery and matrix effect were evaluated at three different levels (LOQ, 6 × LOQ and 30 × LOQ) in triplicate and in within-day conditions.

### 5.4. Control of the Analytical Sequences

For analysis control, samples were divided into analytical sequences and each one of them included at least 8 matrix-matched calibrators in order to obtain a calibration curve for each mycotoxin in each analytical sequence. These calibration curves were used for mycotoxin quantification in the samples analyzed in each analytical sequence. Results were accepted if the calibration curve complied with the criteria of a minimum of six points, a determination coefficient (R^2^) greater than 0.99, and back-calculated concentration for each one of the calibration samples not differed (RE in %) by more than 15% from the nominal value (20% for LOQ level) [34].

In addition, for each mycotoxin in the samples, its identification was carried out based on the presence of both, qualification and quantification, product ions in the chromatogram. Moreover, the ratio (q/Q in %) should not have more than 20% difference with the obtained mean ratio in calibrators of the corresponding sequence. RE in % was calculated as shown in Equation (3):

(3) $$\left(\frac{\frac{q}{Q}\mathrm{in}\;\mathrm{the}\;\mathrm{sample}-\frac{q}{Q}\mathrm{mean}\;\mathrm{value}\;\mathrm{in}\;\mathrm{calibrators}}{\frac{q}{Q}\mathrm{mean}\;\mathrm{value}\;\mathrm{in}\;\mathrm{calibrators}}\right)\times \;100$$

Besides, retention time for the peak of each mycotoxin on the chromatogram should not differ by more than 2.5% from the mean of the retention time for this mycotoxin in the calibrators included in the analytical sequence [74].

### 5.5. Analysis of the Plasma Samples

The 438 samples were once analyzed for mycotoxin presence. After that, some samples were analyzed once again in order to check the quality of the results based on the levels of OTA (see below *Reanalysis*).

Finally, three hundred and forty-six samples were analyzed once again after an enzymatic treatment using a mixture of β-glucuronidase/arylsulfatase enzymes. One hundred and sixty-six samples were from women and 180 from men. These samples were selected taking into account that the remaining plasma volume was enough for plasma treatment and also that the sample had not been submitted to more than two freeze/thaw cycles. This was because during method validation, STER instability was observed after three freeze/thaw cycles [28].

### 5.6. Reanalysis

According to the criteria mentioned in FDA [34], a total of 10% of the samples (40 samples) were reanalyzed in a new sequence including also adequate matrix-matched calibrators in order to prepare simultaneously the corresponding calibration curves for each one of the mycotoxins. During the selection of samples, it was taken into account to include samples from both gender groups (19 from men and 21 from women) and samples with low, medium and high levels of OTA. In order to evaluate the reanalysis, those samples with OTA levels between LOD and LOQ have been considered to be 2 ng/mL (LOQ for OTA). The results obtained after the reanalysis were compared with those obtained in the first analysis, and RE was calculated as it is indicated in Equation (4):

(4) $$\frac{\left[\mathrm{reanalysis}\;\mathrm{level}-\mathrm{original}\;\mathrm{level}\right]}{\mathrm{mean}\;\mathrm{value}}\;\times \;100$$

Following the FDA guideline [34], mycotoxin level in the reanalysis might not have a RE greater than 20% in the 2/3 (67%) of the reanalyzed samples.

After enzymatic treatment, no reanalysis was possible because there was not enough plasma volume.

### 5.7. Statistical Analysis

All statistical analyses were performed using RStudio version 1.2.5019 (Boston, MA, USA). First of all, a descriptive analysis was carried out. A Shapiro–Wilk test studied the normality of the distributions. However, as the hypothesis of normality distribution of the quantitative data was refused, this forced the use of non-parametric statistical methods. Differences between concentration levels for each gender (men and women) were studied using the Wilcoxon Rank Sum (Mann–Whitney). In order to compare mycotoxin levels before and after enzymatic treatment, a Wilcoxon signed-rank test data for non-parametric data was applied. In this statistical analysis, only the samples undergoing the treatment were taken into account and included in the “before enzymatic treatment” group. Differences between age groups (19–39, 40–59, 60–68 years old) were analyzed using a Kruskal–Wallis test. The Spearman correlation coefficient was used to assess the relationship between OTA and OTB levels.

All data above the corresponding LOD were included in the statistical analysis. In the case of obtaining a value lower than the LOD, half of the LOD value was used. The significance of the different statistical tests was set as *p*-value < 0.05 (95% CI).

## Figures and Tables

**Table 1:** q/Q ratios (%) for calibrators and samples for OTA, OTB and STER.

	OTA		OTB		STER
	BE ^a^	AE ^b^	BE	AE	AE
Calibrators	77.2 ± 7.2	66.6 ± 1.9	43.3 ± 1.5	42.2 ± 0.9	86.6 ± 1.7
Samples	75.9 ± 10.1	67.3 ± 3.6	43.5 ± 4.7	42.6 ± 2.3	85.0 ± 4.3

^a^ BE: before enzymatic treatment; ^b^ AE: after enzymatic treatment.

**Table 2:** Retention times for calibrators and samples for OTA, OTB and STER.

	OTA		OTB		STER
	BE ^a^	AE ^b^	BE	AE	AE
Calibrators	15.56 ± 0.28	15.30 ± 0.06	11.31 ± 0.27	11.13 ± 0.06	15.36 ± 0.06
Samples	15.64 ± 0.27	15.45 ± 0.06	11.27 ± 0.21	11.12 ± 0.06	15.38 ± 0.07

^a^ BE: before enzymatic treatment; ^b^ AE: after enzymatic treatment.

**Table 3:** Results obtained in samples without data of gender and age.

Sample	OTA (ng/mL)	OTB (ng/mL)	Sample	OTA (ng/mL)	OTB (ng/mL)
29	2.9		38	4.4	
30	2.4		39	3.1	
31	*1.9* ^a^		348	4.8	*0.5*
32	2.0		406	4.5	
33	*1.3*		407	5.5	
34	*0.7*		408	4.0	
35	*1.5*		409	10.5	
36	*1.2*		410	3.7	
37	*1.5*		411	4.4	

^a^ Number in italics: <LOQ (OTA 2 ng/mL, OTB 1 ng/mL). Empty spaces: values < LOD. LODs: OTA and OTB 0.4 ng/mL.

**Table 4:** Levels of OTA, OTB and STER (ng/mL) found in women plasma samples.

Sample	Age	Before Enzymatic Treatment		After Enzymatic Treatment			Sample	Age	Before Enzymatic Treatment		After Enzymatic Treatment			Sample	Age	Before Enzymatic Treatment		After Enzymatic Treatment		
		OTA	OTB	OTA	OTB	STER			OTA	OTB	OTA	OTB	STER			OTA	OTB	OTA	OTB	STER
40	43	3.2		5.3		1.1	63	62	2.6		5.0	*0.6*	*0.9*	90	49	4.9		5.9	*0.7*	*0.9*
41	65	2.8		5.0	*0.9* ^a^		65	44	2.0		2.4		*0.9*	91	35	*1.6*		n.e.	n.e.	n.e.
42	49	*1.3*		2.6		1.1	66	39	*1.5*		2.6		1.0	93	58	2.4		2.8		1.1
43	49			*1.8*		1.0	68	43	2.4		n.e.	n.e.	n.e.	94	46	2.6		2.8		*0.9*
44	43	4.7		7.2	*0.9*		69	50	4.4		8.6	*0.8*	*0.9*	95	51	*1.8*		2.2		*0.9*
45	63	*0.6*		2.3		*0.9*	70	58	*1.4*		*1.8*		*0.8*	96	68	*1.6*		2.3		*0.9*
47	66	45.7	1.7	n.e.	n.e.	n.e.	73	41	*1.9*		2.0		*0.9*	97	58	2.4		2.2		1.0
48	50	*1.3*		2.7		*0.9*	74	60	2.1		3.7		*0.9*	98	42	2.6		4.7		*0.9*
49	60	*0.7*		2.4		*0.9*	75	41	3.1		5.3	*0.7*	*0.9*	99	49	*1.5*		*1.8*		*0.8*
50	50	2.0		2.1	*0.9*		76	53	*1.6*		n.e.	n.e.	n.e.	101	66	4.8		n.e.	n.e.	n.e.
51	39	*0.5*		2.2		*0.9*	79	44	*0.7*		2.4		*0.8*	102	46	2.2		2.5		*0.8*
52	46	*1.3*		2.4	*0.8*		80	39	2.5		3.0		1.0	103	52	5.4		8.6	*0.8*	*0.8*
53	56	*1.0*		4.5		1.4	81	37	3.6		4.1	*0.6*	*0.7*	104	45	2.4		2.9		*0.9*
54	55	2.6		4.3	*0.8*		82	62	*1.9*		2.3		*0.8*	107	52	2.1		n.e.	n.e.	n.e.
55	47	*1.2*		3.4		1.1	83	30	2.0		2.7		*0.7*	108	53	*1.8*		3.7	*0.5*	*0.8*
56	32	2.5		4.6		1.0	85	51	2.8		2.8		*0.9*	110	48	6.6	*0.5*	8.9	*0.6*	*0.9*
58	55	*1.0*		n.e.	n.e.	n.e.	86	52	2.6		4.0		1.0	111	56	3.5	*0.4*	2.3		*0.9*
59	47	*1.4*		2.6		1.0	87	57	3.8		4.1		*0.9*	112	46	4.5		4.1		*0.9*
60	32	*0.6*		2.6		1.0	88	67	3.5		4.7		1.0	114	60	3.6	*0.4*	2.3		1.0
62	46			2.8		*0.8*	89	42	2.3		2.7		*0.9*	115	51	3.8	*0.4*	2.3	*0.5*	1.0
116	53	3.6	*0.4*	3.9	*0.5*	*0.7*	201	48	2.5	*0.9*	n.e.	n.e.	n.e.	228	61	3.4		2.0		*0.3*
117	48	3.3		2.1	*0.6*	1.4	202	63	2.0	1.0	n.e.	n.e.	n.e.	229	55	*1.5*		*1.2*		*0.4*
118	37	5.9	*0.6*	6.2		*0.9*	203	55	2.6	*0.7*	n.e.	n.e.	n.e.	230	40	*0.9*		*0.9*		*0.5*
119	61	8.3	*0.7*	8.6	*0.6*	1.0	204	45	2.3	*0.7*	n.e.	n.e.	n.e.	231	64	*0.9*		*0.5*		*0.3*
120	53	2.8		n.e.	n.e.	n.e.	205	52	*1.9*		n.e.	n.e.	n.e.	232	56	14.7	*0.7*	13.4	1.1	
121	50	5.3		5.9	*0.5*	*0.9*	206	39	3.3	*0.7*	n.e.	n.e.	n.e.	233	49	2.0		2.0		*0.3*
122	45	2.9		*1.9*		*0.9*	207	64	3.0		n.e.	n.e.	n.e.	234	51	*1.3*		*0.9*		*0.3*
123	45	3.3		*1.9*		*0.8*	208	42	2.2		n.e.	n.e.	n.e.	235	61	*1.1*		*0.9*		*0.5*
124	44	2.9		*1.9*		*0.9*	210	61	2.5		n.e.	n.e.	n.e.	236	48	*1.1*		*1.2*		
125	47	3.1		*1.9*		*0.8*	211	54	*0.8*		*0.7*		*0.5*	237	56	*1.4*		*1.1*	1.1	1.3
126	49	5.1	*0.5*	7.4	*0.7*	1.0	212	68	2.0		*0.9*		*0.2*	238	57	*1.5*	*0.5*	*1.7*		*0.6*
127	66	2.8		2.0		*0.7*	213	62	11.4	*0.7*	17.3	1.3	*0.2*	239	52	*1.4*		*1.3*		*0.6*
128	41	3.7		2.9		*0.9*	214	53	2.1		*1.6*		*0.4*	240	59	10.1	*0.7*	n.e.	n.e.	n.e.
129	49	3.6		2.5		*0.9*	215	39	*1.6*		n.e.	n.e.	n.e.	241	58	*1.6*		*1.6*		*0.4*
195	58	3.5		n.e.	n.e.	n.e.	216	51	2.7		*1.8*		*0.4*	242	56	*1.0*		*1.6*		*0.6*
196	64	3.8	*0.8* ^a^	n.e.	n.e.	n.e.	217	49	*1.3*		*1.6*		*0.4*	243	37	*1.4*		*1.6*		*0.7*
197	55	2.8	*0.8*	n.e.	n.e.	n.e.	218	58	*1.8*		n.e.	n.e.	n.e.	245	57	3.5		2.8		*0.4*
198	54	2.9	*0.8*	n.e.	n.e.	n.e.	225	42	*1.2*		*0.8*		*0.3*	247	58	*0.9*		*1.1*	*0.7*	*0.7*
199	66	4.6	*0.9*	n.e.	n.e.	n.e.	226	61	*0.8*		*0.9*		*0.4*	258	60	*0.8*		*0.7*		*0.4*
200	57	3.9	*0.9*	n.e.	n.e.	n.e.	227	63	*1.3*		*1.4*		*0.5*	264	54			*0.7*		
265	47	*0.8*		*1.2*		*0.4*	299	37	*1.1*		*1.8*		*0.4*	327	57	7.4	*0.5*	8.6	*0.5*	
270	60	*0.5*		*0.7*		*0.4*	300	47	3.1		n.e.	n.e.	n.e.	328	35	2.4		3.3		*0.5*
276	43	*0.9*		n.e.	n.e.	n.e.	301	32	*0.6*		*1.0*		*0.5*	335	62	*1.6*		n.e.	n.e.	n.e.
277	50			*0.8*		*0.7*	303	49	*0.8*		*1.1*		*0.4*	336	65	*1.9*		2.2		*0.6*
278	43	*0.4*		*1.5*		*0.5*	304	63			*0.8*		*0.5*	337	61	*0.5*		*1.0*		*0.6*
279	51	2.5		5.4			305	57	*0.5*		*0.7*		*0.3*	347	50	3.5		2.0		*0.7*
280	60			*0.9*		*0.6*	306	54	2.0		3.1		*0.4*	352	50	6.9		2.7		
281	51			*0.5*		*0.3*	307	56	*0.7*		*1.8*			353	53	3.1				*0.5*
282	40	*1.7*		*1.7*		*0.3*	308	30	*1.1*		*1.5*		*0.6*	364	47	3.2		*1.6*		
283	42						309	56	2.6		2.3		*0.5*	369	46	4.3				*0.5*
284	66	*0.6*		*0.6*		*0.3*	310	65	2.4		3.9			378	55	4.7		*0.7*		*0.4*
285	40	*0.8*		3.0		*0.7*	315	62	*0.5*		*0.9*		*0.4*	379	35	5.1		*1.8*		*0.5*
286	57	4.0		5.4	*0.6*		317	49	*1.7*		*1.9*		*0.5*	380	60	4.2		n.e.	n.e.	n.e.
287	40			*0.4*		*0.3*	318	58	*0.8*		*0.7*		*0.4*	381	48	4.0				*0.5*
290	34	*1.2*		*1.2*		*0.7*	320	67	15.6	*0.6*	8.4			382	48	5.5		2.2		*0.6*
291	63	2.6		4.5		*0.5*	322	40	2.6		*1.5*		*0.4*	383	50	4.1				*0.5*
293	50	3.0		4.0			323	46	*1.8*		2.1		*0.5*	384	54	9.4		5.7	*0.9*	*0.7*
294	38	*1.7*		*1.6*		*0.6*	324	33	*1.9*		*1.5*		*0.4*	385	21	4.6				*0.5*
296	64			2.7		*0.7*	325	52	*0.8*		*1.1*		*0.6*	386	37	3.9				*0.5*
297	59	*1.8*		2.8		*0.9*	326	42	*0.7*		*0.9*		*0.4*	397	54	3.7		*1.8*		*0.6*
399	49	4.3		*0.8*		*0.5*	447	46	2.6				*0.7*	475	52	*1.9*		n.e.	n.e.	n.e.
400	45	5.0		n.e.	n.e.	n.e.	448	55	2.4		*1.7*		*0.8*	476	38	*0.9*		*0.4*		*0.4*
401	61	3.7				*0.4*	449	52	4.3		3.2		1.0	483	42	*1.6*		*1.7*		*0.4*
402	66	3.7		*0.6*		*0.5*	452	41	2.8		n.e.	n.e.	n.e.	484	47	2.3		n.e.	n.e.	n.e.
404	39	4.5		2.6		1.0	453	40	2.9		n.e.	n.e.	n.e.	495	59	3.7		n.e.	n.e.	n.e.
405	41	4.1		*1.9*		*0.8*	454	39	2.7		n.e.	n.e.	n.e.	496	47	2.3		n.e.	n.e.	n.e.
421	57	4.3				0.7	455	37	6.4		n.e.	n.e.	n.e.	518	31	2.3		n.e.	n.e.	n.e.
422	48	3.0		n.e.	n.e.	n.e.	468	41	2.7		*1.1*		*0.3*	519	67	2.3		*0.8*		*0.4*
446	43	2.9		n.e.	n.e.	n.e.	474	52	*1.9*		*1.6*			522	58	3.5		n.e.	n.e.	n.e.

^a^ Number in italics: < LOQ (OTA 2 ng/mL, OTB and STER 1 ng/mL). Empty spaces: values < LOD. n.e.: no enzymatic treatment. LODs: OTA and OTB 0.4 ng/mL, STER 0.2 ng/mL.

**Table 5:** Levels of OTA, OTB and STER (ng/mL) found in men plasma samples.

Sample	Age	Before Enzymatic Treatment		After Enzymatic Treatment			Sample	Age	Before Enzymatic Treatment		After Enzymatic Treatment			Sample	Age	Before Enzymatic Treatment		After Enzymatic Treatment		
		OTA	OTB	OTA	OTB	STER			OTA	OTB	OTA	OTB	STER			OTA	OTB	OTA	OTB	STER
46	48	*1.7* ^a^		n.e.	n.e.	n.e.	249	65	*1.5*		*1.1*		*0.3*	273	57	*1.3*		*1.8*		*0.4*
57	56	*1.4*		n.e.	n.e.	n.e.	250	48	*1.4*		n.e.	n.e.	n.e.	274	32	*0.4*		*0.4*		*0.4*
61	32			2.7		*0.9*	251	59	*0.9*		*1.1*			275	49	*1.9*		*1.6*		*0.4*
64	39	2.8		5.0	*0.8*	*0.8*	252	55	*0.7*					288	53	*1.2*		n.e.	n.e.	n.e.
67	67	*0.7*		n.e.	n.e.	n.e.	253	57	*1.7*		*1.7*		*0.4*	289	53	*0.9*		2.4		
71	57	4.6		3.6	*0.5*	*0.7*	254	52	*0.8*					292	54	*0.6*		*0.9*		*0.4*
72	51	3.0		4.9	*0.8*	1.2	255	51	4.5	*0.4*	4.8	*0.6*		295	65			*0.8*		*0.5*
77	48	2.2		n.e.	n.e.	n.e.	256	48	*0.8*		*0.5*		*0.3*	298	19	4.3		4.6		
78	53	5.0		7.9	*0.6*	*0.9*	257	46	*1.1*		2.1	*0.7*	*0.5*	302	47	*0.8*		2.2		
84	35	*1.7*		3.8	*0.6*	*0.9*	259	47	*1.2*		*1.2*		*0.4*	311	46	*1.9*		2.0		*0.4*
92	34	2.2		n.e.	n.e.	n.e.	260	53	4.7		5.6			312	42	*0.9*		*1.1*		*0.6*
100	55	2.7		n.e.	n.e.	n.e.	261	45	2.1		2.0		*0.5*	313	48	5.6		5.4		
105	51	*1.9*		n.e.	n.e.	n.e.	262	60	*0.5*		*0.8*		*0.5*	314	56	*1.0*		*1.1*		*0.4*
106	56	*1.8*		2.2		*0.8*	263	63	*1.5*		2.7	*0.8*	*0.6*	316	68	*1.0*		*1.4*		*0.4*
109	48	*1.6*		2.5		*0.8*	266	41	*1.6*		7.0	*0.9*	*0.6*	319	41	*1.1*		*1.3*		*0.4*
113	52	3.4		n.e.	n.e.	n.e.	267	27	*0.6*		2.6		*0.4*	321	63	*1.3*		*1.7*		*0.6*
209	46	2.6		n.e.	n.e.	n.e.	268	47	*1.7*		7.6			329	39	*0.9*		2.2		
244	45	2.0		*1.4*		*0.3*	269	34	*1.0*		*1.3*		*0.3*	330	57	3.0		3.1		*0.4*
246	52	*1.3*		*1.5*			271	51	*0.7*		*1.1*		*0.3*	331	42	*1.0*		*1.4*		*0.5*
248	58	5.7		9.7	*0.5*	*0.3*	272	53	*0.7*					332	61	17.7	*0.5*	20.5	*0.6*	
333	44	*1.4*		n.e.	n.e.	n.e.	363	56	3.5		*1.3*		*0.6*	418	45	5.4		3.6		*0.9*
334	40	*1.0*		*1.4*		*0.4*	365	55	3.2		*1.6*		*0.7*	419	35	4.2		*1.4*		*0.5*
338	65	*1.1*		*1.1*		*0.5*	366	57	3.9		*1.3*		*0.7*	420	65	7.1		*1.3*		*0.6*
340	61	3.4	*0.5*	*0.7*		*0.4*	367	50	4.1		*1.6*		*0.6*	423	66	3.4		*0.8*		*0.5*
341	38	3.5	*0.5*	*0.5*		*0.5*	368	47	3.9		*0.7*		*0.8*	424	62	4.1		*0.9*		*0.6*
344	48	3.2				*0.5*	387	31	4.3		*1.1*		*0.6*	425	51	2.6		n.e.	n.e.	n.e.
345	60	3.8		*1.4*		*0.5*	389	54	4.1		*1.2*		*0.7*	426	34	2.7		*0.5*		*0.6*
346	47	4.0		3.0		1.0	390	35	4.4		n.e.	n.e.	n.e.	427	38	2.6		*0.4*		*0.6*
349	53	3.6		*0.5*		*0.5*	391	40	4.1		*1.3*		*0.6*	428	53	3.1		*0.9*		*0.7*
350	36	4.0	*0.5*	*1.4*		*0.4*	393	28	4.2		*1.6*		*0.7*	429	47	2.7		2.0		*0.6*
351	43	4.2		*1.6*		*0.5*	394	39	5.3		3.9	*0.4*	*0.8*	430	37	5.7		3.3		*0.5*
354	48	3.0		*1.9*		*0.2*	395	29	4.2		*1.6*		*0.6*	431	45	2.7				*0.6*
355	45	3.0				*0.4*	396	19	5.7		3.5		*0.8*	432	31	2.8				*0.6*
356	53	3.9		*1.1*		*0.5*	398	58	3.8		*0.9*		*0.6*	433	63	3.1		*1.1*		*0.7*
357	47	3.3				*0.4*	412	52	4.3		*1.9*		*0.6*	434	27	2.2				*0.6*
358	49	5.7		4.0		*0.7*	413	50	6.2		5.4		*0.7*	435	34	2.7		2.7		*0.8*
359	63	3.4				*0.5* ^a^	414	38	4.5		*1.5*		*0.6*	436	37	3.9		*1.4*		*0.6*
360	37	4.0		n.e.	n.e.	n.e.	415	65	4.1		2.7		1.0	437	29	4.0		2.3		*0.6*
361	51	3.2				*0.4*	416	47	4.6		*1.5*		*0.5*	438	47	2.5		2.6		*0.6*
362	39	3.8		*1.0*		*0.5*	417	50	3.5		*0.8*		*0.6*	439	59	2.8		*0.4*		*0.6*
440	23	2.5				*0.6*	469	40	3.5		2.4		*0.6*	494	43	2.9		2.2		*0.5*
441	35	3.4		*0.7*		*0.6*	470	28	2.4	*0.8*	n.e.	n.e.	n.e.	497	32	4.4	*0.5*	3.8		*0.5*
442	40	3.7		*1.8*		*0.5*	471	40	*1.6*	*0.6*	2.0		*0.5*	498	38	3.1		*1.8*		
443	49	2.7		2.7		*0.6*	472	59	*1.2*		*0.9*		*0.6*	499	42	*1.9*		*0.4*		*0.3*
444	67	2.1				*0.5*	473	46	*1.8*		*0.7*			500	66	2.1		n.e.	n.e.	n.e.
445	66	2.2		n.e.	n.e.	n.e.	477	57	*0.7*		*0.8*			501	39	2.3		*1.4*		*0.5*
450	27	4.3		n.e.	n.e.	n.e.	478	41	*0.8*		*0.8*		*0.5*	502	36	3.5		*1.8*		*0.3*
451	48	2.8		n.e.	n.e.	n.e.	479	55	3.1		3.3		*0.7*	503	65	2.3		*1.0*		*0.4*
456	28	3.4		n.e.	n.e.	n.e.	480	50	*1.7*		2.2		*0.3*	504	39	2.1		n.e.	n.e.	n.e.
457	24	3.7		n.e.	n.e.	n.e.	481	22	*1.5*	1.1	*1.8*		*0.6*	505	48	3.6		3.0		*0.4*
458	48	2.7		n.e.	n.e.	n.e.	482	61	*1.2*	*0.6*	*1.4*	*0.6*	*0.7*	506	54	2.5		*1.7*		*0.5*
459	37	5.1		n.e.	n.e.	n.e.	485	33	2.6		3.8	*0.7*	*0.2*	507	50	2.5		*1.4*		*0.4*
460	46	2.3		n.e.	n.e.	n.e.	486	39	*1.2*		*0.7*		*0.2*	508	46	5.5		4.4		
461	44	2.9		n.e.	n.e.	n.e.	487	50	*1.5*		*0.6*		*0.3*	509	53	2.9		*1.5*		*0.4*
462	29	2.6		n.e.	n.e.	n.e.	488	19	*1.2*		*0.8*	*0.7*		510	38	2.2		n.e.	n.e.	n.e.
463	56	5.2		3.4		*0.6*	489	21	*1.2*		*0.7*		*0.4*	511	25	2.9		2.2		*0.4*
464	49	3.5		2.3		*0.6*	490	54	*1.3*		*0.5*		*0.3*	512	56	19.9	*0.9*	23.3	1.1	
465	58	3.0		*1.4*		*0.6*	491	42	2.7		n.e.	n.e.	n.e.	513	61	2.2		*1.3*		*0.4*
466	65	4.9		4.1		*0.6*	492	51	*1.2*		*0.7*		*0.3*	514	63	3.6		2.6		*0.5*
467	50	4.2		5.5		*0.6*	493	44	7.5		10.6	*0.8*	*0.5*	515	35	2.7		3.8		
516	46	3.5		2.7		*0.6*	530	56	5.8	*0.5*	n.e.	n.e.	n.e.	541	47	4.2	*0.5*	2.9		*0.5*
517	44	8.2		n.e.	n.e.	n.e.	531	46	2.5		*0.9*		*0.3*	542	52	2.4		*0.9*		*0.4*
520	57	2.7		2.3			532	39	3.8		2.0		*0.4*	543	63	2.8		*1.5*		*0.4*
521	36	3.3		*1.9*		*0.3*	533	24	2.5		*1.0*		*0.4*	544	50	4.4		3.8	*0.6*	
523	64	2.4		*1.1*		*0.3*	534	44	3.9	*0.5*	*1.9*		*0.3*	545	62	2.1		*1.2*		
524	66	2.7		2.4		*0.6*	535	45	2.6		n.e.	n.e.	n.e.	546	23	*1.9*		*0.5*		*0.3*
525	45	5.8	*0.7*	3.1	*0.6*		536	50	3.8		*1.4*		*0.3*	547	48	2.5		*1.2*		*0.4*
526	52	6.0		3.3		*0.4*	537	46	2.9		*1.6*		*0.6*	548	46	4.0		2.4		*0.4*
527	47	6.2		4.5	*0.6*		538	50	3.2		*1.1*		*0.3*	549	39	2.2		*1.1*		*0.2*
528	40	2.8	*0.5*	*1.8*		*0.3*	539	49	5.5	*0.6*	3.7		*0.4*	550	42	*1.9*		*0.9*		*0.4*
529	57	2.8		*1.5*		*0.4*	540	58	4.1		4.0		*0.4*	551	40	5.6	*0.6*	*0.9*		

^a^ Number in italics: < LOQ (OTA 2 ng/mL, OTB and STER 1 ng/mL). Empty spaces: values < LOD. n.e.: no enzymatic treatment. LODs: OTA and OTB 0.4 ng/mL, STER 0.2 ng/mL.

**Table 6:** Summary of the data obtained for OTA before enzymatic treatment.

Age (Years)	Gender	n	% Positives	Mean ± SD	Median	Max Value	*p*-Value (Age Groups)
19–39	Women	27	100.0	2.49 ± 1.64	2.00	6.4	
	Men	55	98.2	2.99 ± 1.36	2.82	5.7	
	Total	82	98.8	2.82 ± 1.46	2.64	6.4	
40–59	Women	138	94.9	2.69 ± 1.97	2.42	14.7	
	Men	129	100.0	3.08 ± 2.17	2.84	19.9	
	Total	267	97.4	2.88 ± 2.08	2.64	19.9	
60–68	Women	42	92.9	2.77 ± 2.98	2.09	45.7	
	Men	29	96.6	3.05 ± 3.17	2.34	17.7	
	Total	71	94.4	2.89 ± 3.04	2.28	45.7	
19–68	Women	207	95.2	2.68 ± 2.16	2.36	45.7	0.581 ^b^
	Men	213	99.0	3.05 ± 2.15	2.81	19.9	0.403
	n.i. ^a^	18	100	3.35 ± 2.31	3.02	10.5	-
	Total	438	97.3	2.87 ± 2.16	2.60	45.7	0.208 ^b^

^a^ n.i.: gender or age data not indicated. ^b^ The woman sample with the highest OTA level has not been included for statistical comparison.

**Table 7:** Summary of the data obtained for OTB before enzymatic treatment.

Age (Years)	Gender	n	% Positives	Mean ± SD	Median	Max Value	*p*-Value (Age Groups)
19–39	Women	27	7.4	0.64 ± 0.11	0.64	0.7	
	Men	55	9.1	0.58 ± 0.29	0.50	1.1	
	Total	82	8.5	0.60 ± 0.25	0.54	1.1	
40–59	Women	138	10.9	0.45 ± 0.25	0.44	0.9	
	Men	129	7.8	0.43 ± 0.22	0.46	0.9	
	Total	267	9.4	0.45 ± 0.23	0.45	0.9	
60–68	Women	42	19.0	0.71 ± 0.43	0.69	1.7	
	Men	29	10.3	0.53 ± 0.07	0.53	0.6	
	Total	71	15.5	0.67 ± 0.38	0.60	1.7	
19–68	Women	207	12.1	0.53 ± 0.32	0.50	1.7	0.163
	Men	213	8.5	0.48 ± 0.23	0.48	1.1	0.467
	n.i. ^a^	18	5.6	0.49	-	0.5	-
	Total	438	10.0	0.51 ± 0.29	0.49	1.7	0.051

^a^ n.i.: gender or age data not indicated.

**Table 8:** Summary of the data obtained for OTA after enzymatic treatment.

Age (Years)	Gender	n	% Positives	Mean ± SD	Median	Max Value	*p*-Value (Age Groups)
19–39	Women	21	90.5	2.22 ± 1.48	1.76	6.20	
	Men	44	93.2	1.84 ± 1.28	1.53	4.60	
	Total	65	92.3	1.96 ± 1.35	1.60	6.20	
40–59	Women	112	93.8	2.60 ± 2.17	1.98	13.4	
	Men	110	92.7	2.40 ± 2.77	1.62	23.3	
	Total	222	93.2	2.50 ± 2.48	1.86	23.3	
60–68	Women	33	97.0	2.78 ± 3.35	2.01	17.3	
	Men	26	92.3	2.15 ± 3.84	1.23	20.5	
	Total	59	94.9	2.50 ± 3.56	1.36	20.5	
19–68	Women	166	94.0	2.58 ± 2.37	1.99	17.3	0.651
	Men	180	93.3	2.23 ± 2.68	1.53	23.3	0.220
	Total	346	93.6	2.40 ± 2.54	1.75	23.3	0.143

**Table 9:** Summary of the data obtained for OTB after enzymatic treatment.

Age (Years)	Gender	n	% Positives	Mean ± SD	Median	Max Value	*p*-Value (Age Groups)
19–39	Women	21	4.8	0.57	-	0.6	
	Men	44	11.4	0.62 ± 0.15	0.65	0.8	
	Total	65	9.2	0.61 ± 0.14	0.61	0.8	
40–59	Women	112	18.8	0.71 ± 0.18	0.66	1.1	
	Men	110	10.9	0.70 ± 0.18	0.63	1.1	
	Total	222	14.9	0.71 ± 0.18	0.65	1.1	
60–68	Women	33	15.2	0.82 ± 0.27	0.69	1.3	
	Men	26	11.5	0.66 ± 0.12	0.59	0.8	
	Total	59	13.6	0.76 ± 0.23	0.67	1.3	
19–68	Women	166	16.3	0.73 ± 0.20	0.66	1.3	0.434
	Men	180	11.1	0.67 ± 0.16	0.63	1.1	0.903
	Total	346	13.6	0.70 ± 0.18	0.65	1.3	0.498

**Table 10:** Summary of the data obtained for STER after enzymatic treatment.

Age (Years)	Gender	n	% Positives	Mean ± SD	Median	Max Value	*p*-Value (Age Groups)
19–39	Women	21	100	0.68 ± 0.22	0.65	1.0	
	Men	44	88.6	0.53 ± 0.18	0.54	0.9	
	Total	65	92.3	0.58 ± 0.21	0.58	1.0	
40–59	Women	112	83.9	0.70 ± 0.27	0.70	1.4	
	Men	110	81.8	0.51 ± 0.18	0.49	1.2	
	Total	222	82.9	0.61 ± 0.25	0.56	1.4	
60–68	Women	33	87.9	0.58 ± 0.26	0.54	1.0	
	Men	26	92.3	0.52 ± 0.15	0.50	1.0	
	Total	59	89.8	0.55 ± 0.22	0.50	1.0	
19–68	Women	166	86.7	0.68 ± 0.26	0.66	1.4	0.148
	Men	180	85.0	0.51 ± 0.17	0.49	1.2	0.645
	Total	346	85.8	0.59 ± 0.23	0.55	1.4	0.480

**Table 11:** Studies on the presence of OTA in plasma/serum/blood samples in Spain.

Region	Matrix	Total Samples	Positive (%)	LOD (ng/mL)	Mean or Median * and (Range) (ng/mL)	Year/Reference
Navarra	Plasma	438	97.3	0.4	2.99 (<LOD–45.7)	This study
Lleida	Blood	325	100	0.018	0.050 * (0.06–10.92)	2011 [40]
Valencia	Serum	168	100	0.01	1.09 (0.15–5.71)	2010 [41]
Lleida	Blood	279	98.6	0.075	0.86 (0.11–8.68)	2009 [42]
Granada	Plasma	83	72	0.21	0.63 (<0.22–6.96)	2001 [43]
Madrid	Plasma	168	100	0.02	1.192 (0.12–5.58)	1998 [44]
Navarra	Plasma	75	53.3	0.52	0.71 (<LOD–4.0)	1998 [33]

* Median value.

**Table 12:** Studies on plasma/serum/blood levels of OTA worldwide over the last five years.

Country	Matrix	Total Samples	Positive (%)	LOD (ng/mL)	Mean and/or (Range) (ng/mL)	Year/Reference
Sweden	Serum	1096	100	0.014	0.055 (0.05–0.658)	2020 [47]
China	Plasma	147	80.7	0.04	0.29 (0.04–6.59)	2020 [48]
	Plasma	260	27.7	0.04	1.21 (0.312–9.18)	2019 [38]
	Plasma	30	0	0.15	< LOD	2018 [49]
Czech Republic	Serum	50	48	0.04	0.14 (< LOD–0.83)	2019 [50]
Italy	Serum	58	69.2	0.005	(0.01–2.60)	2019 [51]
	Serum	53	76.4	0.08	(< LOD–0.79)	2017 [52]
	Serum	62	54.8	0.025	0.026	2016 [53]
Germany	Blood	16	100	n.i.	0.157 (0.079–0.262)	2019 [54]
	Blood	50	100	0.012	0.204	2017 [39]
	Blood	50	100	0.006	0.211 (0.071–0.383)	2016 [55]
	Blood	50	100	0.005	0.21 (0.071–0.383)	2015 [56]
Portugal	Serum	42	100	0.012	0.76 (0.36–4.99)	2018 [57]
Bangladesh	Plasma	104	100	0.05	0.72 (< LOD–6.63)	2018 [58]
Egypt	Serum	98	81.6	0.2	0.33 (0.20–1.53)	2016 [59]

n.i.: not indicated.

**Table 13:** Presence of OTA in different food matrices in the region of Navarra.

Matrix	Total samples	Positive (%)	LOD (ng/mL)	Mean and/or (range) (ng/mL)	Year/Reference
Cow milk	7	0	0.2	n.d.	2018 [32]
Wine	51	100	0.0032	0.016 (0.005–0.14)	2012 [30]
Beer	31	77	0.012	0.044 (<LOD–0.205)	2005 [61]
Wine	40	50	0.005	(LOD–0.316)	2002 [62]
			**(μg/kg)**	**(μg/kg)**	
Cereals (barley)	123	58	0.013	0.10 (<LOD–3.53)	2012 [29]
Breakfast cereals	46	39	0.062	0.29 (<LOD–1.12)	2011 [63]
Breakfast cereals	21	90	0.066	0.265 (<LOD–0.975)	2005 [61]
Cereals (baby food)	20	70	0.035	0.187 (<LOD–0.740)	2005 [61]
Cereals (wheat, barley, corn)	115	58	0.066	0.219 (<LOD–7.61)	2003 [64]

n.d.: not detected.

## Supplementary Materials

The following are available online at https://www.mdpi.com/2072-6651/12/12/750/s1, Table S1: Examples of the obtained calibration curves. Table S2: Revalidation parameters after enzymatic treatment. Precision and accuracy at three concentration levels (LOQ, 6 × LOQ and 30 × LOQ), calculated as RSD or RE (%). Table S3: Revalidation parameters after enzymatic treatment. Recovery and matrix effect (%RSD). Table S4: OTA reanalysis in plasma samples.

## Abbreviations

15-ADON	15-acetyldeoxynivalenol
3-ADON	3-acetyldeoxynivalenol
ACN	acetonitrile
AFB1	aflatoxin B1
AFB2	aflatoxin B2
AFG1	aflatoxin G1
AFG2	aflatoxin G2
AFM1	aflatoxin M1
AFs	aflatoxins
CI	confidence interval
DAS	diacetoxyscirpenol
DOM-1	deepoxy-deoxynivalenol
DON	deoxynivalenol
EDI	estimated daily intake
EDTA	ethylendiaminetetraacetic acid
EFSA	European Food Safety Authority
EMEA	European Medicines Agency
ESI	electrospray ionization
FDA	Food and Drug Administration
FUS-X	fusarenon-X
HBM	human biomonitoring
IARC	International Agency for Research on Cancer
LC	liquid chromatography
LC-MS/MS	liquid chromatography- mass spectrometry
LOD	limit of detection
LOQ	limit of quantification
ME	matrix effect
NEO	neosolaniol
NIV	nivalenol
OTα	ochratoxin α
OTA	ochratoxin A
OTA-d_5_	ochratoxin A-(*phenyl*-d_5_)
OTB	ochratoxin B
OTC	ochratoxin C
q	transition of qualification
Q	transition of quantification
QqQ	triple quadrupole
RE	relative error of the mean
RSD	relative standard deviation
S/N	signal-to-noise ratio
SRM	selected reaction monitoring
STER	sterigmatocystin
TWI	tolerable weekly intake
ZEA	zearalenone
