# Supplementary material for: Presence of 19 Mycotoxins in Human Plasma in a Region of Northern Spain
Source: Toxins (Basel). 2020 Nov 27;12(12):750. doi: 10.3390/toxins12120750 (PMC7760949; doi:10.3390/toxins12120750)
Supplement: Supplementary file 1 [file toxins-12-00750-s001.pdf]

Supplementary Materials: Presence of 19 Mycotoxins in Human Plasma in a Region of Northern Spain

Beatriz Arce-López, Elena Lizarraga, Ángel Irigoyen and Elena González-Peñas*

Table S1. Examples of the obtained calibration curves.

DOM-1			AFG2			AFM1			AFG1		
ng/mL	B-C (ng/mL)	RE (%)	ng/mL	B-C (ng/mL)	RE (%)	ng/mL	B-C (ng/mL)	RE (%)	ng/mL	B-C (ng/mL)	RE (%)
6.0	6.40	5.66	0.8	0.90	12.91	0.8	0.91	13.22	0.3	0.32	7.18
12.0	10.84	15.51	1.6	1.44	9.72	1.6	1.39	12.85	0.6	0.59	1.61
24.0	22.91	6.77	3.2	3.19	0.30	3.2	3.09	3.30	1.2	1.23	2.09
36.0	35.92	0.23	4.8	4.61	3.89	4.8	4.96	3.24	1.8	1.76	2.23
48.0	49.28	2.67	6.4	6.81	6.34	6.4	7.00	9.34	2.4	2.52	5.08
60.0	64.44	7.39	8.0	7.58	5.29	8.0	7.74	3.29	3.0	2.89	3.63
120.0	119.22	0.65	16.0	15.44	3.47	16.0	15.38	3.87	6.0	5.94	0.92
180.0	176.99	1.67	24.0	24.82	3.43	24.0	24.33	1.39	9.0	9.05	0.50
y = 83.44x − 63.85			y = 626.99x − 27.40			y = 410.34x − 15.98			y = 86.15x − 73.82		
R^2 = 0.9981			R^2 = 0.9969			R^2 = 0.9978			R^2 = 0.9995		
Weight: 1/x			Weight: 1/x			Weight: None			Weight: 1/x		

B-C: back-calculated; RE: relative error.

Table S1. (Continued). Examples of the obtained calibration curves.

AFB2			AFB1			HT-2			OTB		
ng/mL	B-C (ng/mL)	RE (%)	ng/mL	B-C (ng/mL)	RE (%)	ng/mL	B-C (ng/mL)	RE (%)	ng/mL	B-C (ng/mL)	RE (%)
0.3	0.36	18.79	0.2	0.23	14.47	6.0	6.26	4.28	1.0	1.17	17.21
0.6	0.58	3.39	0.4	0.37	8.64	12.0	10.73	10.61	2.0	1.94	3.06
1.2	1.21	0.93	0.8	0.74	7.63	24.0	24.03	0.10	4.0	4.24	6.11
1.8	1.83	1.91	1.2	1.15	4.33	36.0	37.14	3.16	6.0	5.99	0.20
2.4	2.50	4.07	1.6	1.73	8.07	48.0	49.95	4.06	8.0	8.21	2.59
3.0	2.88	3.86	2.0	1.96	1.91	60.0	58.65	2.26	10.0	9.73	2.68
6.0	5.80	3.36	4.0	3.94	1.45	120.0	118.44	1.30	20.0	19.15	4.27
9.0	9.14	1.53	6.0	6.09	1.43	180.0	180.81	0.45	30.0	30.57	1.90
y = 1433.4x − 90.03			y = 2844.47x − 136.89			y = 52.85x − 16.61			y = 747.54x − 241.03		
R^2 = 0.9986			R^2 = 0.9974			R^2 = 0.9995			R^2 = 0.9982		
Weight: None			Weight: 1/x			Weight: None			Weight: None		

B-C: back-calculated; RE: relative error.

Table S1. (Continued). Examples of the obtained calibration curves.

T-2			ZEA			OTA-d5			STER		
ng/mL	B-C (ng/mL)	RE (%)	ng/mL	B-C (ng/mL)	RE (%)	ng/mL	B-C (ng/mL)	RE (%)	ng/mL	B-C (ng/mL)	RE (%)
1.0	1.15	15.07	4.0	4.35	8.72	2.0	2.05	2.70	1.0	1.06	6.43
2.0	1.86	6.84	8.0	7.10	11.25	4.0	4.16	3.99	2.0	1.79	10.39
4.0	4.18	4.59	16.0	-	*	8.0	8.28	3.55	4.0	4.08	2.08
6.0	6.09	1.53	24.0	25.26	5.23	12.0	11.74	2.13	6.0	6.13	2.12
8.0	8.13	1.66	32.0	32.31	0.97	16.0	16.36	2.35	8.0	8.47	5.89
10.0	9.81	1.86	40.0	39.22	1.94	20.0	19.80	0.98	10.0	10.11	1.15
20.0	19.33	3.36	80.0	76.31	4.61	40.0	38.84	2.90	20.0	18.61	6.96
30.0	30.44	1.45	120.0	123.45	2.87	60.0	60.74	1.23	30.0	30.74	2.47
y = 3576x − 935.47			y = 185.95x − 115.93			y = 236.91x − 85.24			y = 737.25x − 72.43		
R^2 = 0.9989			R^2 = 0.9977			R^2 = 0.9992			R^2 = 0.9960		
Weight: None			Weight: 1/x			Weight: None			Weight: None		

B-C: back-calculated; RE: relative error*: not included.

Table S1. (Continued). Examples of the obtained calibration curves.

NIV			DON			FUS-X			NEO		
ng/mL	B-C (ng/mL)	ng/mL	ng/mL	B-C (ng/mL)	RE (%)	ng/mL	B-C (ng/mL)	RE (%)	ng/mL	B-C (ng/mL)	RE (%)
20.4	20.52	6.0	8.7	7.77	10.90	8.7	9.91	13.65	0.8	0.88	10.00
40.8	36.27	12.0	17.4	15.20	12.82	17.4	18.85	8.08	1.6	1.52	4.80
81.6	82.39	24.0	34.9	37.03	6.16	34.9	3377	3.20	3.2	3.13	2.34
122.4	125.52	36.0	52.3	51.64	1.29	52.3	53.50	2.26	4.8	4.98	3.84
163.2	183.19	48.0	69.8	72.18	3.46	69.8	74.23	6.41	6.4	6.56	2.50
204.0	195.47	60.0	87.2	90.09	3.31	87.2	85.78	1.63	8.0	7.22	9.74
408.0	383.88	120.0	174.4	168.36	3.47	174.4	160.07	8.22	16.0	15.24	4.73
612.0	625.17	180.0	261.6	264.05	0.94	261.6	270.22	3.29	24.0	25.26	5.26
y = 4.64x − 18.09			y = 24.5x + 18.42			y = 16.19x − 31.98			y = 181.00x − 16.54		
R^2 = 0.9957			R^2 = 0.9987			R^2 = 0.9943			R^2 = 0.9952		
Weight: None			Weight: None			Weight: None			Weight: 1/x		

B-C: back-calculated; RE: relative error.

Table S1. (Continued). Examples of the obtained calibration curves.

3-ADON			15-ADON			DAS		
ng/mL	B-C (ng/mL)	RE (%)	ng/mL	B-C (ng/mL)	RE (%)	ng/mL	B-C (ng/mL)	RE (%)
1.8	2.08	19.04	2.7	2.77	1.92	0.7	0.84	19.35
3.5	2.98	14.78	5.4	5.41	0.52	1.4	1.23	11.84
7.0	7.15	2.18	10.9	10.64	2.20	2.8	2.62	6.59
10.5	10.62	1.16	16.3	16.71	2.37	4.2	4.23	0.80
14.0	15.08	7.70	21.8	23.15	6.38	5.6	5.54	1.13
17.5	17.35	0.86	27.2	27.83	2.33	7.0	6.97	0.36
35.0	32.81	6.26	54.4	49.70	8.64	14.0	13.36	4.55
52.5	53.70	2.29	81.6	84.11	3.07	21.0	21.91	4.32
$y = 64.22x - 40.31$			$y = 77.18x - 39.69$			$y = 381.06x - 37.92$		
$R^2 = 0.9963$			$R^2 = 0.9941$			$R^2 = 0.9965$		
Weight: None			Weight: None			Weight: 1/x		

B-C: back-calculated; RE: relative error.

Table S2. Revalidation parameters after enzymatic treatment. Precision and accuracy at three concentration levels (LOQ, 6xLOQ and 30xLOQ). Calculated as RSD or RE (%).

Mycotoxin	Precision (%RSD)						Accuracy (%RE)					
	Within-run (n=3)			Between-run (n=9)			Within-run (n=3)			Between-run (n=9)		
	L	M	H	L	M	H	L	M	H	L	M	H
DOM-1	1.96	4.61	11.82	5.04	7.37	6.34	6.89	3.45	6.53	8.80	2.15	4.96
AFG2	6.53	5.31	2.61	7.86	9.83	8.02	1.25	5.90	6.14	7.36	5.02	1.83
AFM-1	5.23	4.20	3.14	8.39	6.98	5.12	3.33	6.11	4.69	6.67	2.43	3.73
AFG1	3.45	7.73	4.05	8.18	9.40	7.26	3.33	0.74	7.89	8.89	5.00	6.93
AFB2	6.37	5.10	1.89	5.20	6.02	8.53	8.89	3.89	8.07	11.48	2.16	3.99
AFB1	5.56	5.12	3.14	6.16	7.64	8.56	10.00	1.67	8.83	14.44	4.63	7.83
HT-2	10.76	4.45	0.83	7.01	12.02	6.87	11.83	5.87	0.94	14.89	3.27	5.93
OTB	3.33	3.32	1.08	4.54	10.05	4.11	4.00	5.78	4.37	7.22	4.54	5.75
T-2	9.67	4.80	5.94	12.08	11.47	13.06	5.00	4.61	9.38	0.44	6.41	3.95
ZEA	6.26	4.68	4.70	11.61	6.97	8.05	1.25	1.21	9.31	6.31	6.18	7.76
OTA-d_5	5.38	6.66	1.76	12.21	11.49	3.93	10.67	3.94	1.54	5.61	6.74	1.78
STER	7.21	7.58	3.90	8.22	14.17	13.20	0.00	5.06	10.37	0.44	0.96	0.29
NIV	12.56	8.62	4.08	14.28	9.82	13.14	4.28	3.52	11.84	0.58	7.72	3.55
DON	7.99	3.02	2.21	10.39	5.08	14.28	11.92	3.02	10.32	9.68	1.04	0.80
FUS-X	11.04	5.17	9.10	11.68	7.41	13.21	8.31	5.51	10.98	10.16	3.69	5.88
NEO	9.80	2.55	2.46	15.77	5.82	10.09	11.25	0.14	7.94	4.22	5.09	2.79
3-ADON	2.91	1.20	1.44	4.71	7.65	9.04	10.19	5.27	3.06	7.85	3.02	3.10
15-ADON	3.84	6.97	2.55	9.36	6.17	6.74	2.35	0.61	3.12	0.05	5.60	3.21
DAS	6.21	3.53	3.67	6.62	12.89	7.86	3.81	4.37	3.51	4.11	4.39	2.91

RSD: relative standard deviation; RE: relative error; L: low level; M: medium level; H: high level.

Table S3. Revalidation parameters after enzymatic treatment. Recovery and matrix effect (%RSD).

	DOM-1	AFG2	AFM-1	AFG1	AFB2	AFB1	HT-2	OTB	T-2	ZEA
	81.17	82.59	89.88	78.77	85.14	93.11	101.06	96.32	103.05	90.43
Recovery	83.31	93.15	88.16	88.16	81.58	86.36	90.20	89.78	87.39	83.02
	94.71	79.23	77.88	76.75	76.27	74.36	78.95	74.55	77.19	68.83
Mean	86.40	84.99	85.31	81.22	81.00	84.61	90.07	86.89	89.21	80.76
RSD (%)	8.43	8.55	7.60	7.50	5.51	11.22	12.27	14.60	14.60	13.59
	71.77	90.95	115.31	88.59	105.34	86.89	104.15	100.03	106.01	89.59
Matrix effect	72.83	92.04	95.24	82.97	100.87	88.63	103.66	103.50	103.37	99.81
	68.89	93.12	101.21	84.42	94.76	88.52	104.60	106.34	107.91	101.36
Mean	71.16	92.04	103.92	85.32	100.32	88.02	104.14	103.29	105.76	96.92
RSD (%)	2.86	1.18	9.92	3.42	5.29	1.11	0.45	3.06	2.16	6.60

RSD: relative standard deviation.

Table S3. (Cont.). Revalidation parameters after enzymatic treatment. Recovery and matrix effect (%RSD).

	OTA-d_5	STER	NIV	DON	FUS-X	NEO	3-ADON	15-ADON	DAS
	89.39	90.84	79.30	92.56	97.47	85.43	90.45	92.58	93.47
Recovery	87.85	74.42	76.53	94.66	94.95	82.45	84.85	90.08	96.01
	70.23	56.50	65.39	78.96	68.25	78.37	81.63	76.07	73.87
Mean	82.49	73.92	73.74	88.73	86.89	82.08	85.64	86.24	87.79
RSD (%)	12.91	23.23	9.99	9.61	18.64	4.32	5.22	10.32	13.80
	99.26	66.58	92.23	91.98	86.31	94.26	103.53	89.56	104.18
Matrix effect	100.81	72.69	92.52	79.94	83.70	86.22	87.31	86.18	99.12
	102.67	83.41	94.25	91.49	102.85	101.05	94.94	99.36	111.89
Mean	100.91	74.23	93.00	87.81	90.95	93.84	95.26	91.70	105.06
RSD (%)	1.69	11.48	1.17	7.76	11.42	7.91	8.52	7.46	6.12

RSD: relative standard deviation.

Table S4. OTA reanalysis in plasma samples.

Women Samples	OTA (ng/mL)			Men Samples	OTA (ng/mL)		
	First analysis	Reanalysis	RE (%)		First analysis	Reanalysis	RE (%)
47	45.7	42.3	7.7	46	< LOQ	< LOQ	0.0
58	< LOQ	2.4	18.2	57	< LOQ	< LOQ	0.0
68	2.4	< LOQ	17.4	67	< LOQ	< LOQ	0.0
76	< LOQ	< LOQ	0.0	77	2.2	< LOQ	11.3
91	< LOQ	< LOQ	0.0	92	2.2	< LOQ	7.7
101	4.8	4.0	17.2	100	2.7	< LOQ	30.1
107	2.1	< LOQ	2.5	105	< LOQ	< LOQ	0.0
120	2.8	< LOQ	32.6	113	3.4	< LOQ	52.1
215	< LOQ	< LOQ	0.0	250	< LOQ	2.1	6.8
218	< LOQ	< LOQ	0.0	288	< LOQ	< LOQ	0.0
240	10.1	8.6	15.6	333	< LOQ	< LOQ	0.0
300	3.1	2.7	12.4	360	4.0	< LOQ	67.1
335	< LOQ	< LOQ	0.0	390	4.4	< LOQ	75.0
380	4.2	< LOQ	71.2	425	2.6	< LOQ	24.6
400	5.0	2.5	64.7	445	2.2	< LOQ	9.5
422	3.0	< LOQ	40.3	470	2.4	< LOQ	19.8
446	2.9	< LOQ	37.4	500	2.1	< LOQ	4.9
475	< LOQ	2.2	8.2	510	2.2	< LOQ	8.6
496	2.3	< LOQ	13.5	530	5.8	3.8	41.7
518	2.3	< LOQ	14.4				
522	3.5	2.5	33.4				

RE: relative error; LOQ: limit of quantification (included in the statistical study as 2 ng/mL).
